# G protein regulatory network shapes magnitude and kinetics of behavioral responses in an engineered opioid receptor model

**DOI:** 10.1016/j.jbc.2026.111425

**Published:** 2026-04-02

**Authors:** Deziree L. Coleman, Rachel J. Ren, Karla J. Opperman, Elizabeth X. Kwan, Kirill A. Martemyanov, Brock Grill

**Affiliations:** 1 Norcliffe Foundation Center for Integrative Brain Research, Seattle Children's Research Institute, Seattle Children's Hospital, Seattle, Washington, USA; 2 Department of Pharmacology, University of Washington School of Medicine, Seattle, Washington, USA; 3 Department of Pediatrics, University of Washington School of Medicine, Seattle, Washington, USA; 4 Department of Physiology and Biophysics, University of Miami Medical School, Miami, Florida, USA

**Keywords:** behavioral genetics, *C. elegans*, fentanyl, G protein, GPCR, mu-opioid receptor, MOR, opioid, RGS

## Abstract

G protein-coupled receptors (GPCRs) control essential neuronal functions. One GPCR with prominent effects on the nervous system and animal behavior is the mu-opioid receptor (MOR). GPCRs mediate their effects by engaging a gamut of G proteins, which are inhibited by Regulators of G protein signaling (RGS). At present, how different RGS proteins regulate the magnitude and temporal kinetics of G protein signaling to affect behavior remains unclear. Here, we use an engineered cross-species *Caenorhabditis elegans* model of MOR signaling (tgMOR) to test how multiple RGS proteins shape MOR signaling and behavioral responses to opioids. Our results indicate opioid-induced effects on locomotor behavior in tgMOR are primarily mediated by Gαo and are modified by opposing Gαq action. We further delineate that EGL-10 (RGS7) is a primary RGS that modulates the magnitude of MOR-meditated responses. In a differential effect, EAT-16 (RGS9) and its regulator RSBP-1 (R7BP) principally influence the timing of behavioral response onset. Thus, a multi-layered RGS network is required to shape the magnitude and kinetics of MOR signaling and ensuing behavioral responses to opioids. The G protein regulatory network revealed here might also have broader implications for other Gαo/i-coupled receptors.

G protein-coupled receptors (GPCRs) control a vast number of cellular and organismal functions. Within the nervous system, GPCR signaling is important for both neuromodulation and neurotransmission that shapes animal behavior (1, 2, 3). GPCRs mediate their effects by engaging a gamut of G proteins each endowed with specific properties. G proteins are in turn controlled by Regulators of G protein signaling (RGS) that negatively modulate GPCR signaling. A broad swath of RGS proteins have been identified and shown to influence GPCR signaling within and outside the nervous system. Evidence from cell-based studies indicates that a range of RGS players can regulate an individual G protein (4, 5, 6, 7). However, we still know relatively little about how different RGS proteins function together *in vivo* to shape the magnitude and temporal kinetics of G protein signaling. While individual RGS proteins have been shown to affect GPCR-mediated behaviors, how a complement of multiple RGS players influence GPCR signaling to impact behavior remains relatively poorly understood (1). Cross-species engineered GPCR signaling systems combined with organismal models present a valuable opportunity to examine how RGS networks shape GPCR signaling to influence behavior.

One GPCR with particularly prominent roles in the nervous system is the μ-opioid receptor (MOR). MOR is a key GPCR found in the central and peripheral nervous system that mediates the clinically beneficial and harmful effects of opioids (8, 9, 10). Opioids remain one of the most effective treatments for pain despite severe negative side effects including tolerance, abuse and dependence (11, 12). Therapeutic strategies aimed at dissociating the harmful effects of opioids (such as dependence and tolerance) from manipulation of their beneficial actions like analgesia will require that we achieve a deep understanding of the regulatory principles and players that shape MOR signaling (10, 13). At present, how RGS players converge on MOR signaling to affect behavioral outcomes remains unclear.

G proteins coupled to MOR are the principal mediators of opioid signaling (14). MOR is known to signal *via* the inhibitory class of Gαi/o, but the contributions of individual members of this G protein class to opioid responses are not well understood particularly in behavior (15, 16, 17, 18, 19). Prior *in vivo* studies using heterozygous Gαo KO mice showed that Gαo mediates morphine antinociception in hot plate but not tail flick pain models (20). Studies with knock-in mice that carry an RGS insensitive Gαo mutant (resulting in increased Gαo signaling) indicated that both supraspinal and spinal nociceptive responses to morphine were increased (21). At present, it is unclear how prominent a player Gαo is in MOR signaling, and we know little about the evolutionary importance of Gαo in mediating MOR signaling.

We previously generated a transgenic cross-species *Caenorhabditis elegans* model of MOR signaling (tgMOR) that displays robust behavioral responses to opioids (22, 23, 24, 25). The tgMOR model exogenously expresses mammalian MOR broadly in the nervous system, which imbues *C. elegans* with opioid-induced effects on locomotor behavior that do not occur in the absence of tgMOR. This indicates that exogenous mammalian MOR engineered into neurons hijacks presently unknown, endogenous signaling machinery to generate opioid drug responses. In this study, we show that a highly conserved *C. elegans* Gαo protein called G protein o-alpha subunit (GOA)-1 mediates locomotor behavioral responses to fentanyl in our engineered, cross-species model of MOR signaling. Thus, our findings indicate that Gαo is likely to be an ancient evolutionarily conserved mediator of MOR signaling and behavioral responses to opioids.

RGS proteins are important negative modulators of G proteins and GPCR signaling that have been studied from *C. elegans* through mammals (26). When G proteins are activated, they bind GTP and trigger downstream signaling. Over time, RGS proteins bind activated G proteins and accelerate GTPase activity thereby switching off G protein signaling (7, 27, 28, 29, 30, 31, 32). In the mammalian nervous system, several RGS proteins that are selective for Gαi/o are implicated in modulating opioid responses (4, 7, 31, 33, 34). This includes RGS6, RGS7, and RGS9 as well as R7BP which forms a negative regulatory complex with RGS7. RGS9 negatively modulates MOR signaling in mouse KO models, and both RGS9 and RGS7 regulate behavioral responses to the rewarding effects of morphine (35, 36, 37). While multiple RGS proteins affect opioid-induced behavior, we presently do not understand whether different RGS proteins shape the temporal kinetics and magnitude of behavioral responses.

Here, we evaluate how both egg laying defective (EGL)-10 (RGS6/7) and eating abnormal (EAT)-16 (RGS9) shape the effects of fentanyl on the tgMOR *C. elegans* model. We combine genetics, CRISPR engineering, and automated, unbiased approaches for monitoring locomotor behavior to examine how each RGS player influences the magnitude and kinetics of MOR signaling in response to fentanyl. Our results indicate that EGL-10 (RGS6/7) primarily affects the magnitude of the fentanyl response. EAT-16 (RGS9) and its regulatory binding partner R-seven binding protein homolog (RSBP)-1 (R7BP) principally modulate the timing of opioid-induced effects on locomotor responses. Taken as a whole, we have identified a multi-layered RGS network that tunes the magnitude and temporal kinetics of Gαo signaling in an engineered opioid model.

## Results

### Opioid-induced behavioral responses of tgMOR C. elegans have both magnitude and temporal components

**Figure 1:**
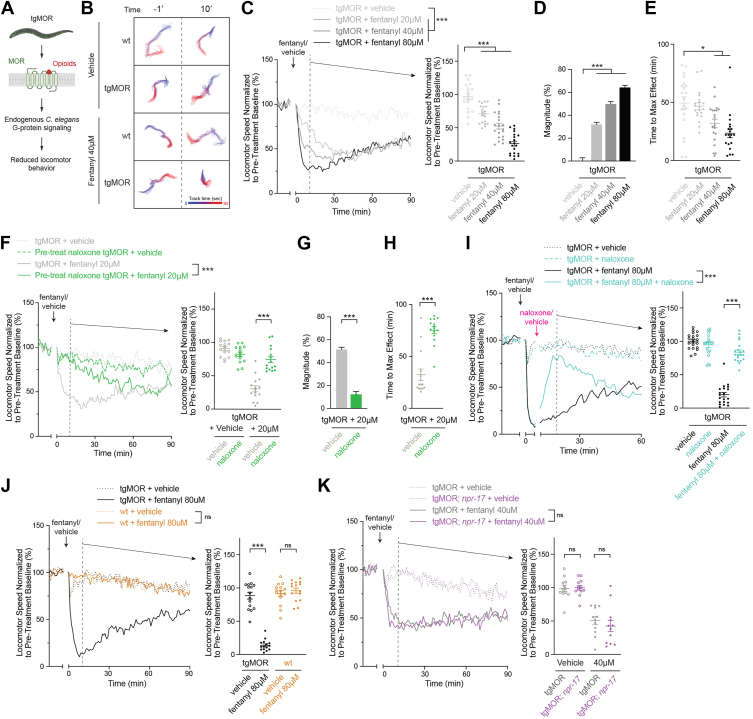
**TgMOR *C. elegans* display dose-sensitive behavioral responses to fentanyl that are inhibited by naloxone and absent in non-transgenic animals.***A*, schematic of tgMOR *C. elegans* where mammalian MOR is transgenically expressed and engages unknown, endogenous G protein signaling machinery to generate opioid-induced effects on locomotor behavior. *B*, computationally automated tracking of locomotion in liquid using MWT showing reduced locomotor speed of tgMOR *C. elegans* treated with 40 μM fentanyl. Plots show 30 s traces for individual wt or tgMOR animals prior to (−1′) and 10 min post-treatment (10′) with 40 μM fentanyl or vehicle. *C*, quantitation shows tgMOR *C. elegans* display stronger reductions in locomotion with increasing fentanyl dose. Shown are MWT plots of average locomotor speed (*left*) and expanded quantitation (*right*) at set time point 10 min after fentanyl or vehicle treatment. *Arrow* indicates fentanyl or vehicle application. *D*, quantitation of percent magnitude shows stronger fentanyl effects with increasing dose. *E*, time to maximum effect for fentanyl is shorter with increasing dose. *F*–*H*, pretreating tgMOR with naloxone (20 μM) for 30 min reduce effects of fentanyl (20 μM). *I*, naloxone (20 μM) reverses effects of fentanyl (40 μM) on tgMOR animals. *J,* high-dose fentanyl (80 μM) impairs locomotion of tgMOR *C. elegans* but not wt non-transgenic animals. *K*, fentanyl effects on tgMOR animals are not altered in tgMOR; *npr-17* mutants. *C, F, I, J and K,plots* (*solid lines, left side*) represent mean speed of tracked animals (4 animals/well, five wells per genotype per experiment and 3–4 independent experiments for all genotypes and treatments). *Dots (right side)* represent single wells tracked (4 animals/well), *lines* represent average for all wells, and *error bars* are SEM. Plots are normalized to 10-min baseline locomotor speed prior to treatment. Significance for plots (*left*, *genotype annotations*) was tested using two-way ANOVA with *post hoc* Bonferroni correction for multiple comparisons, and set time point comparisons (*right*) were tested using one-way ANOVA with Bonferroni correction. *D and G,bars* represent average for all wells and *error bars* are SEM. *E and H,dots* represent single wells tracked, *lines* represent average for all wells, and *error bars* are SEM. *D and E,* significance tested using one-way ANOVA with Bonferroni correction. *G and H,* significance tested using Student's *t* test. ∗∗∗*p* < 0.001, ∗*p* < 0.05, ns = not significant. MWT, Multi-Worm Tracker.

We previously generated a *C. elegans* model of opioid signaling and behavior by transgenically expressing the mammalian μ-opioid receptor pan-neuronally in the *C. elegans* nervous system (22, 23) (Fig. 1*A*). This engineered cross-species opioid model, which we refer to as tgMOR, displays robust behavioral sensitivity to opioid drug treatment in high-intensity locomotor assays where *C. elegans* are swimming in liquid. To date, we have not fully examined both magnitude and temporal components of opioid behavioral profiles.

To address this, we used an unbiased, automated behavioral tracking suite, Multi-Worm Tracker (MWT), which we previously adapted to *C. elegans* pharmaco-behavioral assays with opioids and other drugs (23, 38). We use MWT to monitor *C. elegans* swimming speed for 10 min prior to drug application, apply fentanyl or vehicle, and then continue recording locomotion for a specified amount of time. MWT acquires locomotor data for individual animals across 20 wells (4 animals per well). MWT plots of individual animals showed that treating tgMOR *C. elegans* with 40 μM fentanyl for 10 min resulted in robust reduction in locomotor swimming speed (Fig. 1*B*). Movies also showed that tgMOR animals treated with fentanyl for 10 min were paralyzed, and lacked responsivity to touch stimulus (Movies S1 and S2). Quantitation of MWT data indicated that fentanyl across multiple doses (20–80 μM) significantly reduces locomotor speed of tgMOR compared to vehicle (Fig. 1*C*). Magnitude of locomotor responses were evaluated with two quantitative metrics: 1) expanded quantitative analysis at a set time point (10-min post-fentanyl) (Fig. 1*C*, right panel), and 2) quantitation of percent magnitude for the main sensitivity window (1–30 min post-fentanyl) (Fig. 1*D*). We observed dose-dependent effects for both metrics of magnitude, which were significantly stronger with increasing fentanyl dose from 20-80 μM fentanyl compared with vehicle (Fig. 1, *C* and *D*). As an independent behavioral component, we quantitatively evaluated time to maximum effect which was significantly faster for 40 and 80 μM fentanyl compared to vehicle (Fig. 1*E*).

Next, we further evaluated how magnitude and time of behavioral responses to fentanyl (MOR agonist) are affected by application of the MOR antagonist, naloxone. First, we pretreated tgMOR *C. elegans* with naloxone for 30 min, which blocked both magnitude and temporal effects of fentanyl (Fig. 1, *F*–*H*). Second, we applied naloxone after fentanyl treatment, which also reversed the effects of fentanyl on locomotion (Fig. 1*I*). We also found that wt non-transgenic *C. elegans* that do not express mammalian MOR fail to respond to the highest dose of fentanyl tested (Fig. 1*J*), which is consistent with prior findings (22). We further evaluated whether a previously identified endogenous kappa-like opioid receptor, NeuroPeptide Receptor family (*npr*)*-17* (39, 40, 41, 42), contributes to tgMOR responses to opioids. We found fentanyl sensitivity in tgMOR; *npr-17* mutants was not significantly different from tgMOR animals, and locomotion was not affected in vehicle treated tgMOR; *npr-17* mutants compared to vehicle treated tgMOR controls (Fig. 1*K*). These results indicate that NPR-17 does not affect swimming locomotion or opioid-induced effects on locomotion in tgMOR *C. elegans*.

Collectively, our findings support several important points. 1) In the tgMOR *C. elegans* model, dose-sensitive effects on the magnitude of locomotor response can be quantitatively evaluated. 2) Opioid-induced responses also display a temporal component that is dose sensitive. 3) Finally, both the magnitude and kinetics of fentanyl responses are affected by naloxone. Importantly, these expanded quantitative metrics for tgMOR locomotor responses allow us to begin examining how mediators and regulators of MOR signaling contribute to different components of opioid-induced behaviors.

### Endogenous Gαo mediates opioid-induced locomotor responses in tgMOR C. elegans

**Figure 2:**
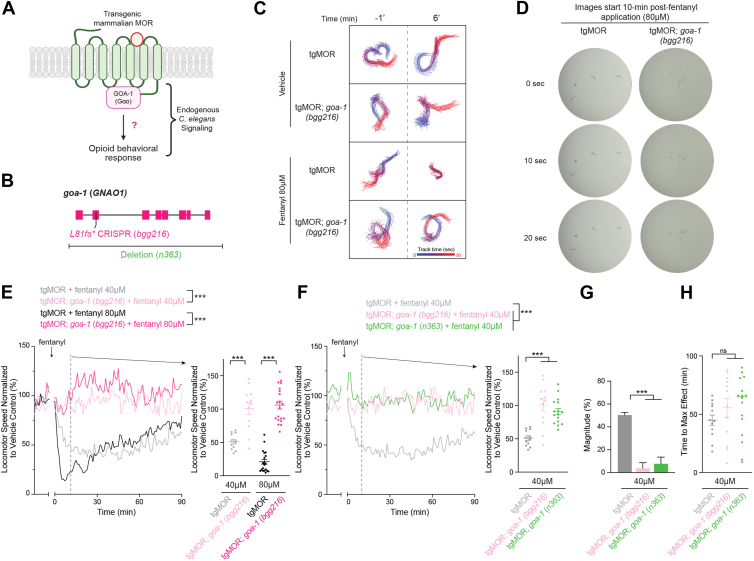
**GOA-1 Gαo mediates effects of fentanyl on tgMOR *C. elegans*.***A,* schematic depicts GOA-1 (GNAO1) G protein that potentially mediates MOR signaling and opioid-induced effects on locomotor behavior in tgMOR *C. elegans*. *B*, *goa-1* gene diagram with CRISPR engineered frameshift (*L81fs∗, bgg216*) and deletion allele (*n363*). *C*, example of MWT plots for single animals showing fentanyl effects on tgMOR are impaired in tgMOR; *goa-1* mutants. Shown are plots over 30 s before treatment (*upper panels*) and 6 min post-vehicle or 80 μM fentanyl treatment (*lower panels*). *D*, still-frame images of single wells with four animals showing effects of fentanyl are impaired in tgMOR; *goa-1* mutants. Images are shown every 10 s following 5 min of treatment with 80 μM fentanyl. *E*, quantitation shows tgMOR; *goa-1* mutants display impaired sensitivity to 40 μM and 80 μM fentanyl compared to tgMOR animals. Shown are MWT plots of average locomotor speed normalized to vehicle control (*left*) and expanded quantitation (*right*) at set time point 10 min after fentanyl treatment for indicated genotypes. *Arrow* indicates fentanyl application. *F*, quantitation shows two tgMOR; *goa-1* mutant alleles (*bgg216* and *n363*) display impaired sensitivity to 40 μM fentanyl. *G*, magnitude of fentanyl effects shows two tgMOR; *goa-1* mutants (*bgg216* and *n363*) display impaired responses to fentanyl compared to tgMOR controls. *H*, time to maximum effect for fentanyl shows no significant difference between tgMOR; *goa-1* mutants and tgMOR controls. *E and F,plots* (*solid lines, left side*) represent mean speed of tracked animals (4 animals/well, four wells per genotype per experiment and 3 to 4 independent experiments for all genotypes and treatments). *Dots* (*right side*) represent single wells tracked (4 animals/well), *lines* represent average for all wells, and *error bars* are SEM. Significance for plots (*left*) was tested using two-way ANOVA with *post hoc* Bonferroni correction, and set time point comparisons (*right*) were tested using one-way ANOVA with Bonferroni correction. *G,bars* represent average for all wells and *error bars* are SEM. *H,dots* represent single wells tracked, lines represent average for all wells, and *error bars* are SEM. *G and H,* significance tested using one-way ANOVA with Bonferroni correction. ∗∗∗*p* < 0.001, ∗∗*p* < 0.01, ns = not significant. MWT, Multi-Worm Tracker.

At present, it is unknown how the endogenous G protein signaling network shapes opioid responses in the tgMOR *C. elegans* model (Fig. 2*A*). We previously used large-scale, unbiased forward genetics with tgMOR *C. elegans* to identify genetic regulators of MOR signaling and opioid-induced behavior (22, 23). As part of our forward genetic approach, we isolated a tgMOR mutant with reduced behavioral responses to fentanyl, which we identified as having a frameshift in *goa-1 (L81fs∗)*. GOA-1 is the sole *C. elegans* Gαo protein that is orthologous to human GNAO1 (Alliance of Genome Resources; Wormbase). Conservation is striking with *C. elegans* GOA-1 displaying 82.2% identity and 94.6% conservation with human GNAO1 (Fig. S1). To validate this *goa-1* mutation, we used CRISPR editing to introduce the identical frameshift mutation into tgMOR *C. elegans* to generate tgMOR; *goa-1* (*L81fs∗*, *bgg216*) mutants (Fig. 2*B*). MWT track plots, movies, and still frame-images showed that individual tgMOR; *goa-1* mutants did not reduce swimming locomotion in response to fentanyl (Fig. 2, *C* and *D*; Movies S3 and S4). Thus, effects of fentanyl on tgMOR *C. elegans* were strongly impaired in the absence of Gαo.

Before proceeding to further quantitative studies, we took several considerations into account for tgMOR; *goa-1* mutants. Consistent with prior findings on GOA-1 in *C. elegans* (43), tgMOR; *goa-1* mutants had reduced body size (Fig. 2*D*; Movies S3 and S4). Initial analysis of tgMOR; *goa-1* mutants using MWT also indicated that they displayed hyperactive raw locomotor speed at baseline prior to drug addiction, which is similar to prior observations on *goa-1* mutants including those performed with MWT (Fig. S2*A*) (43, 44). As a result, we performed layers of quantitative analysis using MWT by normalizing tgMOR; *goa-1* mutants for body size (Fig. S2*A*), normalizing data to baseline locomotor speed (Fig. S2*B*), and normalizing data to vehicle treated tgMOR; *goa-1* mutants (Fig. 2*E*). In all cases, our results indicated that the effects of fentanyl at 40 μM or 80 μM were strongly impaired in tgMOR; *goa-1* mutants (Fig 2*E*; Fig. S2, *A*–*D*). These findings suggest that GOA-1 could be a prominent mediator of MOR signaling.

To further validate this concept, we tested an independent *goa-1* deletion allele, *n363* (Fig. 2*B*). Similar to tgMOR; *goa-1 (bgg216)* mutants, tgMOR; *goa-1* (*n363*) mutants also had strongly impaired responses to 40 μM fentanyl (Figs. 2*F*; S2, *E* and *F*). Quantitative analysis indicated that the magnitude of fentanyl response in both tgMOR; *goa-1* mutants was significantly reduced (Fig. 2, *F* and *G*). Time to maximum effect in tgMOR; *goa-1* mutants were not significantly changed compared to tgMOR controls (Fig. 2*H*). This is consistent with the magnitude of fentanyl responses being so substantially impaired in tgMOR; *goa-1* mutants that time is not a particularly informative metric, nonetheless, we present this for clarity and consistency with later parts of this study.

These results with multiple, independent genetic tools indicate that GOA-1 is the primary G protein that mediates MOR signaling and locomotor responses to opioids in the tgMOR *C. elegans* model.

### Magnitude of MOR-mediated locomotor responses are primarily restrained by EGL-10 RGS

Prior large-scale cell-based studies indicate that members of the R7 RGS family, which includes RGS6, RGS7 and RGS9 (45), have prominent selectivity for Gαo (7, 46). Here, we sought to further understand the role of the R7 RGS family in our tgMOR model, where behavioral responses are mediated by GOA-1 (Gαo).

**Figure 3:**
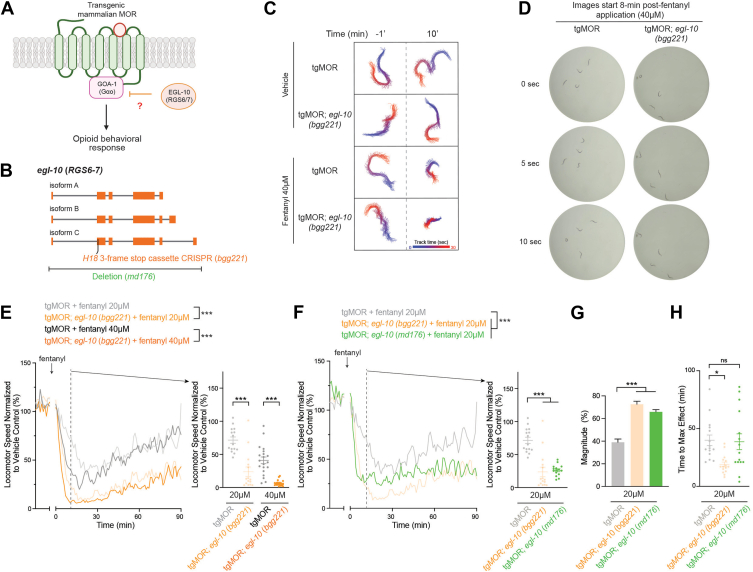
**EGL-10 RGS negatively regulates magnitude of locomotor responses to fentanyl.***A*, schematic depicts EGL-10 (RGS6/7) which potentially regulates MOR signaling and locomotor behavior in response to opioids. *B*, *egl-10* gene diagram with CRISPR engineered 3-frame stop (*H18stop*, *bgg221*) and deletion allele (*md176*). *C*, example of MWT plots for single animals showing tgMOR; *egl-10* mutants are hypersensitive to fentanyl. Shown are plots of tgMOR; *egl-10* mutant and tgMOR control over a 30 s timeframe before (*upper panels*) and 10 min post-vehicle or 40 μM fentanyl treatment (*lower panels*). *D*, still-frame images of single wells with four animals showing tgMOR; *egl-10* mutants have increased effects with 40 μM fentanyl treatment for 8 min. *E*, quantitation shows tgMOR; *egl-10* mutants display increased sensitivity to 20 μM and 40 μM fentanyl. Shown are MWT plots of average locomotor speed normalized to vehicle control (*left*) and expanded quantitation (*right*) at set time point 10 min after fentanyl treatment. *F*, quantitation shows two tgMOR; *egl-10* mutant alleles (*bgg221* and *md176*) display stronger sensitivity to 40 μM fentanyl. *G*, magnitude of fentanyl effects is increased in two tgMOR; *egl-10* mutants (*bgg221* and *md176*) compared to tgMOR control animals. *H*, time to maximum effect for fentanyl is inconsistently affected in tgMOR; *egl-10* mutants. *E and F,plots* (*solid lines, left side*) represent mean speed of tracked animals (4 animals/well, five wells per genotype per experiment and 3–4 independent experiments for all genotypes and treatments). *Dots* (*right side*) represent single wells tracked (4 animals/well), *lines* represent average for all wells, and *error bars* are SEM. Significance for plots (*left*) was tested using two-way ANOVA with *post hoc* Bonferroni correction, and set time point comparisons (*right*) were tested using one-way ANOVA with Bonferroni correction. *G,bars* represent average for all wells and *error bars* are SEM. *H, dots* represent single wells tracked, *lines* represent average for all wells, and *error bars* are SEM. *G and H,* significance tested using one-way ANOVA with Bonferroni correction. ∗∗∗*p* < 0.001, ∗*p* < 0.05, ns, not significant; MWT, Multi-Worm Tracker.

We started by evaluating EGL-10 (RGS6/7), which was previously shown to regulate GOA-1 in *C. elegans* (47, 48) (Fig. 3*A*). To test this candidate, we used CRISPR engineering to insert a 3-frame stop cassette into *egl-10* to generate tgMOR; *egl-10 (bgg221)* (Fig. 3*B*). MWT plots, movies, and still-frame images showed that 40 μM fentanyl has stronger effects on tgMOR; *egl-10* mutants than tgMOR control animals (Fig. 3, *C* and *D*; Movies S5 and S6). For further quantitative analysis with MWT, locomotor data was normalized to speed of vehicle treated tgMOR; *egl-10* mutants (Fig. 3*E*), or baseline locomotor speed prior to fentanyl administration (Fig. S3, *A* and *B*). Our results indicated that tgMOR; *egl-10* mutants are hypersensitive to 20 μM and 40 μM fentanyl showing significantly stronger reductions in locomotor speed compared to tgMOR control animals (Figs. 3, *E* and *F*; S3, *A*–*D*). To further test the role of EGL-10 in opioid responses, we generated an independent tgMOR; *egl-10* mutant using a deletion allele, *md176* (Fig. 3*B*). We observed increased responses to 20 μM fentanyl with more severe reductions in locomotor speed for tgMOR; *egl-10* (*md176*) mutants compared to tgMOR controls (Figs. 3*F*; S3, *E* and *F*).

Next, we performed expanded analysis to further examine how magnitude and temporal kinetics of opioid responses are altered in tgMOR; *egl-10 (bgg221)* and tgMOR; *egl-10* (*md176*) mutants. Our results indicated that the magnitude of the response was significantly greater in both tgMOR; *egl-10* mutants (Fig. 3, *F* and *G*). We observed an inconsistent effect on time to maximum effect for our tgMOR; *egl-10* mutants suggesting that EGL-10 plays a more modest role on temporal kinetics (Fig. 3*H*).

These results support two conclusions. 1) Results with two independent alleles demonstrate that tgMOR; *egl-10* mutants are hypersensitive to fentanyl. This indicates that the EGL-10 RGS negatively regulates MOR signaling. Because RGS proteins negatively regulate G proteins, EGL-10 is likely to inhibit GOA-1 (Gαo) which is the primary mediator of MOR signaling in the tgMOR model. 2) While both the magnitude and kinetics of fentanyl responses are enhanced in tgMOR; *egl-10* mutants, a particularly prominent effect occurs for magnitude of behavioral response.

### EAT-16/RSBP-1 RGS complex primarily regulates kinetics of opioid responses

**Figure 4:**
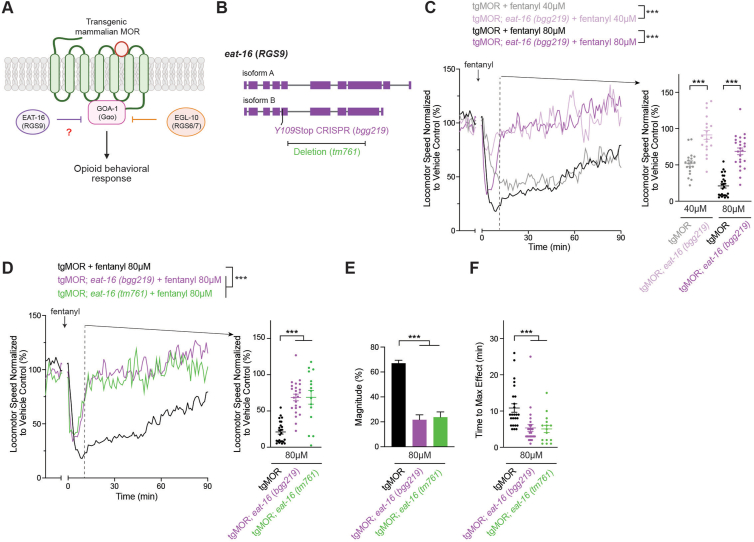
**EAT-16 RGS restrains temporal kinetics of locomotor responses to fentanyl.***A*, schematic depicts EAT-16 (RGS9) which potentially regulates MOR signaling and locomotor behavior in response to opioids. *B, eat-16* gene diagram with CRISPR engineered stop (*Y109Stop, bgg219*) and deletion allele (*tm761*). *C*, quantitation shows tgMOR; *eat-16* mutants display increased sensitivity in time of response to 40 μM and 80 μM fentanyl as well as reduced magnitude of response. Shown are MWT plots of average locomotor speed normalized to vehicle control (*left*) and expanded quantitation (*right*) at set time point 10 min after fentanyl treatment. *D*, quantitation shows two tgMOR; *eat-16* mutant alleles (*bgg219* and *tm761*) display increased sensitivity in time of response to 80 μM fentanyl. *E,* magnitude of fentanyl effects are reduced in both tgMOR; *eat-16* mutants (*bgg219* and *tm761*) compared to tgMOR control animals. *F,* time to maximum effect for fentanyl is faster in tgMOR; *eat-16* mutants compared to tgMOR controls. *C and D,plots* (*solid lines, left side*) represent mean speed of tracked animals (4 animals/well, five wells per genotype per experiment and 3 to 5 independent experiments for all genotypes and treatments). *Dots* (*right side*) represent single wells tracked (4 animals/well), *lines* represent average for all wells, and *error bars* are SEM. Significance for plots (*left*) was tested using two-way ANOVA with *post hoc* Bonferroni correction, and set time point comparisons (*right*) were tested using one-way ANOVA with Bonferroni correction. *E,bars* represent average for all wells and *error bars* are SEM. *F, dots* represent single wells tracked, *lines* represent average for all wells, and *error bars* are SEM. *E and F,* significance tested using one-way ANOVA with Bonferroni correction. ∗∗∗*p* < 0.001. MWT, Multi-Worm Tracker.

**Figure 5:**
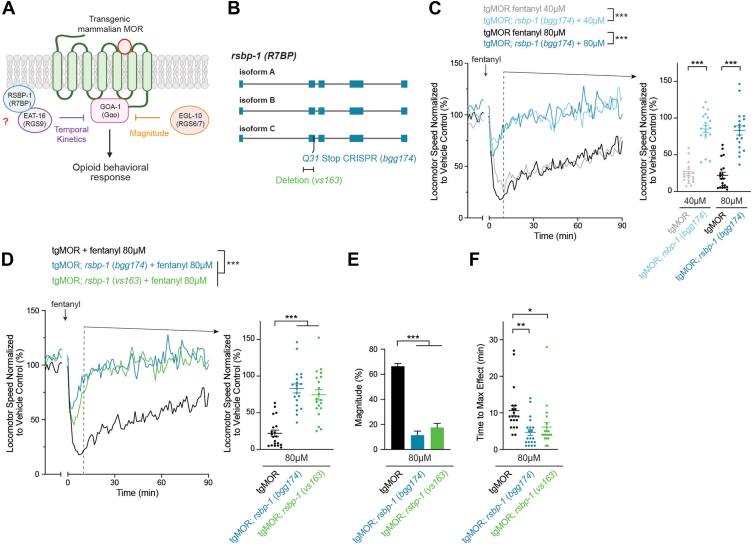
**RSBP-1 restrains temporal kinetics of locomotor responses to fentanyl.***A*, schematic depicts RSBP-1 (R7BP), which forms an RGS complex with EAT-16, and potentially regulates MOR signaling and locomotor behavior in response to opioids. *B*, *rsbp-1* gene diagram with CRISPR engineered stop (*Q31Stop, bgg174*) and deletion allele (*vs163*). *C*, quantitation shows tgMOR; *rsbp-1* mutants display increased sensitivity in time of response to 40 μM and 80 μM fentanyl as well as reduced magnitude of response. Shown are MWT plots of average locomotor speed normalized to vehicle control (*left*) and expanded quantitation (*right*) at set time point 10 min after fentanyl treatment. *D*, quantitation shows two tgMOR; *rsbp-1* mutant alleles (*bgg174* and *vs163*) display increased sensitivity in time of response to 80 μM fentanyl. *E,* magnitude of fentanyl effects are reduced in both tgMOR; *rsbp-1* mutants (*bgg174* and *vs163*). *F,* time to maximum effect for fentanyl is faster in tgMOR; *rsbp-1* mutants compared to tgMOR controls. *C and D,plots* (*solid lines, left side*) represent mean speed of tracked animals (4 animals/well, five wells per genotype per experiment and four independent experiments for all genotypes and treatments). *Dots* (*right side*) represent single wells tracked (4 animals/well), *lines* represent average for all wells, and *error bars* are SEM. Significance for plots (*left*) was tested using two-way ANOVA with *post hoc* Bonferroni correction, and set time point comparisons (*right*) were tested using Student's *t* test. *E, bars* represent average for all wells and *error bars* are SEM. *F, dots* represent single wells tracked, *lines* represent average for all wells, and *error bars* are SEM. *E and F,* significance tested using one-way ANOVA with Bonferroni correction. ∗∗∗*p* < 0.001, ∗*p* < 0.01, ∗*p* < 0.05. MWT, Multi-Worm Tracker.

Interestingly, our unbiased forward genetic screen also isolated mutants for another member of the R7 RGS family, EAT-16 (RGS9), as well as its protein complex partner RSBP-1 (R7BP) (Figs. 4*A* and 5*A*). This might initially appear incongruent as EAT-16 is orthologous to mammalian RGS9 and RSBP-1 is orthologous to mammalian R7BP. However, prior studies in *C. elegans* have shown that EAT-16 forms a functional complex with RSBP-1 (48, 49, 50). Thus, while the preference of RSBP-1 (R7BP) for RGS9 *versus* RGS7 varies across models, the overall concept of R7BP functioning in an RGS regulatory complex remains similar.

To test the role of EAT-16 further, we used CRISPR editing to generate the same early-stop mutation in *eat-16* (*Y109∗, bgg219*) that was isolated in our forward genetic screen, which is likely to generate a molecule null allele (Fig. 4*B*) (22). Quantitative studies using MWT showed that tgMOR; *eat-16 (bgg219)* mutants displayed a significantly altered response to fentanyl compared to tgMOR control animals at both 40 μM and 80 μM fentanyl (Figs. 4*C*; S4, *A*–*D*). To further test EAT-16 for effects on locomotor responses to fentanyl, we generated tgMOR; *eat-16* mutants using an independent deletion allele, *tm761.* Similar to tgMOR *eat-16 (bgg219)* mutants, tgMOR; *eat-16 (tm761)* mutants showed significantly altered fentanyl responses (Figs. 4*D*; S4, *E* and *F*). In our initial quantitative analysis, both tgMOR; *eat-16* mutants displayed faster temporal responses to fentanyl and recovered more quickly. This suggested they were hypersensitive to opioids, but the effect might principally be occurring *via* an effect on time. To test this further, we evaluated how magnitude and temporal kinetics were affected in tgMOR; *eat-16 (bgg219)* and tgMOR; *eat-16 (tm761)* mutants. Our results indicate that while magnitude of response to fentanyl was reduced for both alleles (Fig. 4, *D* and *E*), we detected significantly faster time to maximum effect (Fig. 4*F*). These results indicate that tgMOR; *eat-16* mutants are hypersensitive to fentanyl, but hypersensitivity occurs primarily *via* effects on temporal kinetics of the locomotor response and not magnitude.

Next, we evaluated RSBP-1 for effects on behavioral responses to fentanyl (Fig. 5*A*). To do so, we CRISPR edited the same early-stop mutation from our forward genetic screen into *rsbp-1 (Q31∗, bgg174*), which generates a likely molecular null (Fig. 5*B*) (22). Similar to tgMOR; *eat-16* mutants, tgMOR; *rsbp-1 (bgg174)* mutants were hypersensitive to fentanyl showing a faster time of fentanyl action, increased time to maximum effect, and more rapid recovery from fentanyl (Figs. 5, *C*–*F*; S5, *A*–*D*). We also observed reduced magnitude of fentanyl effects for tgMOR; *rsbp-1 (bgg174)* mutants. Similar outcomes occurred with tgMOR; *rsbp-1 (vs163)* mutants carrying an independent *rsbp-1* deletion allele (Figs. 5, *B*, *D*–*F*; S5, *E* and *F*).

Taken together, our observations support several conclusions. 1) Results with multiple independent alleles for both tgMOR; *eat-16* and tgMOR; *rsbp-1* indicate they have similar opioid behavioral sensitivity profiles, which is consistent with EAT-16 and RSBP-1 forming a complex. 2) Fentanyl has faster temporal effects and recovery occurs more rapidly in both tgMOR; *eat-16* and tgMOR; *rsbp-1* mutants compared to wt tgMOR animals. This indicates the EAT-16/RSBP-1 complex negatively regulates MOR-GOA-1 signaling to shape the temporal kinetics of locomotor responses to opioids. However, reduced magnitude of opioid responses is also observed in these mutants, which suggests EAT-16/RSBP-1 potentially influences another G protein. Prior *C. elegans* studies evaluating EAT-16 outside the context of opioid signaling suggest this could possibly be Gαq (50). 3) Finally, our findings demonstrate that multiple layers of RGS machinery, which includes EGL-10 and EAT-16/RSBP-1, negatively regulate MOR signaling to shape the magnitude and temporal kinetics of behavioral responses to opioids in tgMOR *C. elegans*.

### Gαq gain of function opposes MOR-Gαo signaling

Prior cell-based studies have shown that MOR signaling *via* Gαo/i is opposed by Gαq signaling (51, 52). Results from *C. elegans* also indicate that GOA-1 Gαo signaling is opposed by EGL-30 Gαq signaling during locomotion on solid media and egg-laying behaviors in the absence of opioids and engineered MOR (26, 50, 53). Therefore, we hypothesized Gαo and Gαq signaling might functionally oppose one another to influence opioid behavioral responses of tgMOR *C. elegans*.

**Figure 6:**
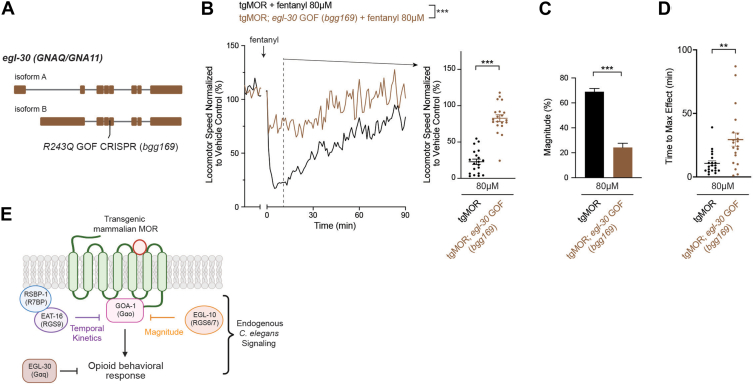
**Constitutively activating EGL-30 Gαq reduces locomotor responses to fentanyl.***A, egl-30* gene diagram with CRISPR engineered gain-of-function mutation *(R243Q GOF, bgg169)*. *B*, quantitation shows tgMOR; *egl-30* GOF mutants display weaker sensitivity to 80 μM fentanyl. Shown are MWT plots of average locomotor speed normalized to vehicle control (*left*) and expanded quantitation (*right*) at set time point 10 min after fentanyl treatment. *C,* magnitude of fentanyl effects is weaker in tgMOR; *egl-30* GOF mutants compared to tgMOR control animals. *D,* time to maximum effect for fentanyl is increased in tgMOR; *egl-30* GOF mutants compared to tgMOR controls. *E,* proposed endogenous *C. elegans* G protein regulatory network that imbues tgMOR *C. elegans* with sensitivity to opioids, and that shapes the magnitude and temporal kinetics of opioid-induced effects on locomotor behavior. *B,plots* (*solid lines, left side*) represent mean speed of tracked animals (4 animals/well, five wells per genotype per experiment and four independent experiments for all genotypes and treatments). *Dots* (*right side*) represent single wells tracked (4 animals/well), *lines* represent average for all wells, and *error bars* are SEM. Significance for plots (*left*) was tested using two-way ANOVA, and set time point comparisons (*right*) were tested using Student's *t* test. *D,bars* represent average for all wells and *error bars* are SEM. *D,dots* represent single wells tracked, *lines* represent average for all wells, and *error bars* are SEM. *C and D,* significance tested using Student's *t* test. ∗∗∗*p* < 0.001, ∗∗*p* < 0.01. MWT, Multi-Worm Tracker.

*C. elegans* EGL-30 is the sole ortholog for two human Gαq proteins, GNA11 and GNAQ (Alliance of Genome Resources; Wormbase). To test the role of EGL-30 (Gαq), we CRISPR edited a gain-of-function (GOF) mutation into *egl-30* (*R243Q, bgg169*) that constitutively activates the G protein by uncoupling RGS proteins and reducing GTP hydrolysis (Fig. 6*A*) (54, 55). We did not evaluate *egl-10* loss-of-function mutations because they are lethal (56). Quantitative studies using MWT showed that tgMOR; *egl-30 GOF (bgg169)* mutants have significantly decreased locomotor responses to 80 μM fentanyl compared to tgMOR control animals (Figs. 6*B*; S6, *A* and *B*). Expanded quantitative analysis showed that tgMOR; *egl-30 GOF* mutants had reduced magnitude (Fig. 6, *B* and *C*) and increased time to maximum effect (Fig. 6*D*). While tgMOR; *goa-1* loss-of-function mutants showed a total loss of fentanyl responsiveness (Fig. 2), tgMOR; *egl-30 GOF* mutants had a more modest reduction in fentanyl responsiveness (Figs. 6, *B*–*D*; S6, *A* and *B*). Overall, our findings support an interesting model: behavioral responses of tgMOR *C. elegans* to opioids are mediated by MOR-Gαo signaling, and modulated by a genetic regulatory network composed of multiple RGS proteins and EGL-30 Gαq signaling (Fig. 6*E*).

## Discussion

We previously used tgMOR as a tractable cross-species genetic model for large-scale forward genetic studies that identified evolutionary conserved receptor systems that regulate MOR signaling and opioid behavioral responses from *C. elegans* through rodents (22, 23). Importantly, tgMOR *C. elegans* is an organismal model where genetic regulatory effects on MOR signaling occur in an intact, native neuronal context. Here, we present our findings that demonstrate two RGS proteins EGL-10 (RGS6/7) and EAT-16 (RGS9) tune the magnitude and temporal kinetics of MOR signaling and locomotor behavior in response to opioids. We have also shown that mammalian MOR engineered into the *C. elegans* nervous system engages endogenous Gαo as a primary mediator of MOR signaling. Finally, our results indicate that Gαq and Gαo signaling functionally oppose one another to shape opioid effects on locomotion. Thus, we combine an engineered whole-animal MOR signaling system, *in vivo* pharmaco-behavioral assays, and a range of genetic strategies to decipher a G protein regulatory network that shapes the magnitude and kinetics of behavioral sensitivity to opioids (Fig. 6*E*).

Early cell-based studies initially showed MOR can signal *via* Gαo (18, 19, 57). Subsequent studies in rodents indicated Gαo mediates a subset of behavioral responses to opioids. First, heterozygous Gαo KO mice were found to display alterations in analgesic responses to morphine (20). Second, anti-sense oligonucleotide studies in rats showed Gαo plays a role in hyperalgesia caused by sub-analgesic fentanyl doses (58). Finally, Gαo gain-of-function studies demonstrated that thermal nociception is increased in a knock-in mouse model carrying an RGS insensitive Gαo mutant (21). Despite this important progress, the role of Gαo in behavioral responses to opioids has remained under-studied. Our results with an independent orthogonal MOR signaling model now emphasize the importance of Gαo in MOR signaling and opioid-induced behavioral responses. Interestingly, the effects of fentanyl are so strongly reduced in tgMOR; *goa-1* mutants that it is clear GOA-1 Gαo is a primary mediator of MOR signaling in our non-canonical whole-animal model. Historically, *C. elegans* GOA-1 was considered the ortholog of both mammalian Gαo and Gαi (59). However, more recent bioinformatic analysis indicates GOA-1 is likely to be the sole primary ortholog of Gαo, while GPA-4 and GPA-16 are orthologous to mammalian Gαi (Alliance of Genome Resources; Wormbase). While our findings demonstrate Gαo mediates MOR signaling in the tgMOR *C. elegans* model, we have not ruled out the possibility that Gαi might also play a role in our model. Our results combined with prior findings in cell-based studies and rodents indicate that Gαo is likely to be an evolutionarily ancient mechanism mediating MOR signaling, and potentially a more prominent mediator of opioid-induced behaviors than presently appreciated. Whether this is the case or not awaits further studies on Gαo in mammalian circuitry that is modulated by MOR signaling.

Genetic results from our tgMOR *C. elegans* model have further revealed that a multi-layered RGS profile shapes the magnitude and temporal kinetics of MOR signaling and locomotor responses to fentanyl. We have found that EGL-10 (RGS6/7) acts as an inhibitory RGS mechanism that can influence the magnitude of opioid responses (Fig. 3). The EAT-16 (RGS9)/RSBP-1 (R7BP) RGS complex also inhibits MOR signaling. However, fentanyl hypersensitivity in tgMOR; *eat-16* and tgMOR; *rsbp-1* mutants is primarily driven by effects on temporal kinetics of locomotion rather than magnitude (Figs. 4 and 5). Prior mammalian cell-based and *in vivo* studies showed that RGS6, RGS7 and RGS9 restrict GPCR and MOR signaling (7, 46). However, these studies did not mechanistically address how different RGS proteins tune MOR signaling to shape the magnitude and temporal kinetics of opioid sensitivity. Thus, our findings could provide a new conceptual framework for future efforts aimed at examining how RGS mechanisms affect the magnitude and kinetics of GPCR signaling and behavior in other models.

Because our results indicate engineered MOR signals heavily through the GOA-1 Gαo (Fig. 2), our findings on RGS proteins are most likely explained by the simple model that both EGL-10 and the EAT-16/RSBP-1 complex inhibit MOR/GOA-1 signaling (Fig. 6*E*). Importantly, outcomes from our behavioral studies are supported by results from orthogonal systems on several levels. 1) Previous large-scale cell-based signaling studies have demonstrated that mamalian RGS6, RGS7 and RGS9 are the top RGS modulators of Gαo (7). 2) Earlier cellular studies specifically focused on RGS9 also indicated that it negatively regulates MOR (36). 3) Rodent behavioral studies have further shown that RGS7 (*C. elegans* EGL-10), RGS9 (EAT-16), and R7BP (RSBP-1) regulate opioid reward and analgesic responses (4, 33, 37). 4) Finally, our results indicate that genetically activating Gαq reduces the effects of fentanyl on tgMOR *C. elegans* (Fig. 6). This is consistent with previous cell-based results that showed Gαq signaling opposes MOR-mediated opioid effects (51, 52, 60), and prior findings from *C. elegans* showing EGL-30 Gαq signaling opposes GOA-1 Gαo to affect locomotion and egg-laying behaviors in the absence of engineered MOR signaling (26, 48, 50, 53, 61). Furthermore, our results are consistent with prior observations from cell-based approaches and the tgMOR *C. elegans* model that showed the Gαq coupled receptor GPR139 (*C. elegans* FRPR-13) negatively regulates MOR signaling (22, 51). Overall, our findings indicate that an evolutionarily conserved, multilayered RGS profile and Gαq restrict MOR signaling and opioid-induced behavioral responses in tgMOR *C. elegans*. Because we are using an engineered *C. elegans* tgMOR model, it is plausible that the G protein regulatory principles we have identified here are not unique to MOR. In fact, it is quite reasonable the conceptual findings from our study could have broader implications for a range of Gαo/i coupled receptors in other systems.

Prior studies in *C. elegans* have shown that EAT-16 (RGS9) regulates Gαq signaling (48, 50, 53). In contrast, mammalian cell-based studies have indicated that RGS9 inhibits Gαo but not Gαq during MOR signaling (7, 36, 62). While this might initially appear as a discrepancy in how RGS9 modulates G protein signaling in different models, we do not think this is the case. While our results align more with findings from mammalian cell-based studies, we think this is reasonable as we have engineered mammalian MOR into *C. elegans*. As a result, our engineered cross-species GPCR system re-orients endogenous RGS/G protein signaling relationships to reflect what occurs with mammalian players and a strongly driven Gαo system. Consistent with this possibility, we have found that tgMOR mutants for the *eat-16/rsbp-1* complex display differing effects on the kinetics and magnitude of opioid responses. Our results indicate EAT-16/RSBP-1 complex inhibition of GOA-1 leads to opioid hypersensitivity that presents principally as kinetic effects (*i.e.,* faster inhibition and recovery of the locomotor response following opioid treatment). However, a bi-functional role for EAT-16/RSBP-1 simultaneously restricting EGL-30 Gαq signaling might also be occurring, which would provide an explanation for why we also observe reduced magnitude of opioid responses in tgMOR; *eat-16* and tgMOR; *rsbp-1* mutants. As a result, it's possible that RGS regulation of G proteins could be more plastic and sensitive to levels of GPCR/G protein signaling, rather than being solely driven by the specificity of individual RGS proteins for particular G proteins.

Our findings also have implications for how the G protein regulatory network we have identified might affect synaptic transmission and neuronal activity in the tgMOR model. Prior studies found that GOA-1 reduces presynaptic neurotransmission and Ca2+ activity in *C. elegans* (61, 63, 64), and results from rodents indicate loss of RGS7 affects neuronal excitability (37). This suggests opioid engagement of MOR/GOA-1 signaling in tgMOR *C. elegans* might result in reduced presynaptic activity or more general reductions in neuronal activity. Because MOR is expressed pan-neuronally in our model it is possible locomotor effects might occur due to broad effects of opioids across the nervous system, or due to more specific effects on the motor neurons.

Our findings indicate the *C. elegans* tgMOR model facilitates genetic studies with opioids using high-intensity, liquid locomotor assays as a simple but robust pharmaco-behavioral readout. We have found that tgMOR engineered into the *C. elegans* nervous system imbues *C. elegans* with opioid sensitivity by engaging endogenous Gαo signaling through GOA-1. MOR-Gαo signaling is modulated by an inhibitory network composed of EGL-10, EAT-16/RSBP-1 and Gαq that restricts opioid-induced behavior. Because all these inhibitory players are highly conserved between *C. elegans* and mammals, our results demonstrate that engineering a mammalian GPCR into the *C. elegans* nervous system engages an ancient, conserved inhibitory regulatory network for Gαo.

## Experimental procedures

### C. elegans strains

Animals were maintained using standard procedures. The following transgenic strain was used: *bggIs79* [P_rgef-1_FLAG::mouse MOR; P_ttx-3_RFP] X. The following mutant and CRISPR engineered alleles were used: *npr-17*(*bgg329* [3-frame stop cassette CRISPR]) III, *goa-1*(*bgg216* [L81fs CRISPR]) I, *goa-1*(*n363*) I, *egl-10*(*bgg221* [3-frame stop cassette CRISPR]) V, *egl-10(md176)* V, *eat-16*(*bgg219* [Y109Stop CRISPR]) I, *eat-16*(*tm761*) I, *rsbp-1*(*bgg174* [Q31Stop CRISPR]) I, *rsbp-1*(*vs163*) I, *egl-30*(*bgg169* [R243Q CRISPR]) I. All strains were grown at 20 °C for at least two generations prior to executing high-intensity swimming locomotor experiments. Mutants, CRISPR alleles and transgenes used for specific experiments are listed in Table S1. Primers used for strain genotyping are listed in Table S2. CRISPR reagents are listed in Table S3. Microinjection conditions used to generate strains are shown in Table S4. All CRISPR engineered mutations were verified by sequencing.

### CRISPR/Cas9 engineering

CRISPR alleles were engineered *via* direct injection of Cas9 ribonucleoprotein complexes with *dpy-10* co-CRISPR. Complexes were formed by mixing recombinant Cas9 protein, tracrRNA (IDT), and crRNA (IDT) at 37 °C for 15 min, followed by addition of ssODN repair template (IDT). CRISPR-Cas9 ribonuclear complexes were injected into tgMOR (*bggIs79*) animals; individual edited alleles were isolated by PCR-based genotyping, and confirmed by sequencing. STOP alleles were designed using a STOP-in cassette (65), which was inserted into the earliest exon that would affect all protein isoforms. All CRISPR targeting sequences and repair templates are listed in Table S3.

### Fentanyl behavioral assays with MWT

MWT was used to quantitatively monitor high-intensity locomotor swimming behavior as described previously (23, 38). In brief, worms were synchronized by picking L4 animals 20 to 24 h prior to MWT assays. Young adult animals were picked into the lid of a 96-well plate with each well containing four animals. Lid wells contained 20 μl of vehicle (M9 + 0.01% tween-20). MWT was used to record animal swimming speed for 10-min baseline. 20 μl fentanyl was added to lid wells at 2X concentration to achieve final desired concentration. Swimming speed was recorded for up to 90 min. Analysis of locomotor speed was performed using a custom script, with speed defined as the movement speed of the animal centroid in mm/s. For mutants such as *goa-1, rsbp-1* and *eat-16* where animals are small or body morphology was altered, MWT acquisition parameters and analysis were normalized accordingly for body size and morphology. Data were analyzed and graphed using Prism (https://www.graphpad.com).

We note that we use relatively high concentrations of opioid drugs in tgMOR *C. elegans* compared to primary cultured neurons, cell-based assays or *in vitro* experiments for several reasons. 1) *C. elegans* is a metazoan organism that has an active metabolism across multiple tissues and cell types that can process and metabolize foreign substances and small molecules (66). 2) Nematodes including *C. elegans* have a complement of xenobiotic detoxification enzymes and transporters that affect drug efficacy (67, 68, 69, 70). As a result, exogenously applied molecules are not likely to accumulate as effectively in *C. elegans*, and higher concentrations of drugs are often required than used in other organisms and cellular assays (66). 3) Finally, the cuticle of *C. elegans* is resistant to small molecules and foreign substances (66, 71). As a result, it is relatively common to use drugs at ∼10 to 100 fold or higher concentrations in *C. elegans* than used in cellular assays.

### Naloxone behavioral assays with MWT

MWT was used to quantify locomotor responses to fentanyl as noted above. For experiments where animals were pretreated with naloxone (Fig. 1*D*), young adult worms were picked into 20 μl of naloxone for 30 min prior to application of fentanyl. MWT was used to record animal swimming speed for the last 10 min of naloxone exposure prior to adding fentanyl to calculate baseline locomotion. 20 μl of fentanyl was added to lid wells at 2X concentration. Swimming speed was recorded for up to 90 min. Naloxone concentration remained consistent throughout the experiment.

For experiments where naloxone was applied post-fentanyl application (Fig. 1*E*), young adult worms were picked into 20 μl of vehicle (M9 + 0.01% tween-20). MWT was used to record swimming speed for 10 min. Next, 20 μl fentanyl or vehicle (M9 + 0.01% tween-20) was added to lid wells at 2X concentration to achieve final desired concentration. MWT was used to record swimming speed for 5 min. Subsequently, 10 μl of naloxone or vehicle (M9 + 0.01% tween-20) was added to wells. We continued to record swimming speed for up to 90 min. Data were analyzed and graphed using Prism (https://www.graphpad.com).

### Generation of MWT single animal tracking plots

Animals were cultured, mounted for recording and monitored using MWT as noted above. Plots represent a single animal in a well. Single animals were tracked for plots to increase resolution of locomotor tracking and prevent overlap of tracking plots which can occur with multiple animals in a well. MWT plots of individual animals moving over 30 s time intervals were generated using a separate custom written script.

### C. elegans movies and time stamps with opioid treatmen*t*

Movies of *C. elegans* in liquid were captured on a Zeiss Stemi 305 stereoscope with Axiocam 208 color camera attachment, or using an iPhone 14 Pro Max with LabCam for iPhone 14 Pro Max adaptor. *C. elegans* were recorded in plates set up as noted above for fentanyl locomotor assays with MWT. In movies, we show two adjacent wells using 40 μl assay buffer or 40 μl of fentanyl and four animals per well. Liquid locomotion was captured for 10 min. Movies were annotated and clipped to subsets noted in supplemental movie legends using Adobe Premiere Pro. For time stamps (Figs. 2*D*, and 3*D*), movies were viewed using Adobe Premiere Pro, and individual image frames were chosen every 5 to 10 s at the indicated time points and exported as JPEG files. Individual movie frames were viewed in Adobe Photoshop and cropped to single well size.

### Statistical analysis and locomotion data normalization parameters

For MWT assays, high-intensity locomotion was recorded on plates with 4 to 5 wells per genotype and treatment, and four animals per well. Data were collected from at least three independent biological experiments from separately cultured plates of animals on different days for all drug or vehicle treatments and genotypes.

In MWT line plots (*e.g.,* Fig. 1*C*, left portions of two-part figure panels), solid lines represent average speed of all animals monitored by MWT (48–80 total animals per genotype and treatment). Data for each genotype and treatment was recorded using MWT and analyzed and presented as follows: 1) Raw speed as a readout, where data acquisition parameters were only adjusted for body size or morphology as needed based on genotype (*e.g.,* Fig. S2*A*). 2) Speed normalized to baseline, where speed was normalized to the average speed of a genotype over a 10-min baseline without drug or vehicle treatment (*e.g.,* Figs. S2*A*; 1*C*). 3) Vehicle control normalized data, where a given genotype was recorded on the same plate with either vehicle or drug treatment (*e.g.,* Fig. 2, *E* and *F*). Data was then normalized to the corresponding vehicle control and presented.

For initial evaluation of magnitude of responses, we performed expanded quantitative analysis at a set time point (*e.g.,* Fig. 1*C*, right portions of two-part figure panels), dots represent average for single wells tracked (4 animals/well), lines represent average for all wells tracked, and error bars are SEM.

To further quantify magnitude (*e.g.,* Fig. 1*G*), we calculated magnitude as a percentage of maximum possible reduction in locomotor speed between 1 to 30 min sensitivity window where effect on magnitude occurred using the following formula:

$$\frac{magnitude\;\left(\%\right)=\left(\text{maximum}\;\text{area}\;\text{above}\;\text{curve}\;1-30\;\min\right)-\left(\text{tgMOR}\;\text{or}\;\text{tgMOR}\;\text{mutant}\;\text{area}\;\text{under}\;\text{curve}\;1-30\;\min\right)}{\text{maximum}\;\text{area}\;\text{above}\;\text{curve}\;1-30\;\min}*100$$

Bar graphs represent average percent magnitude for all wells and error bars are SEM. Area under the curve was calculated using Prism software (https://www.graphpad.com).

For quantitation of time to maximum effect (*e.g.,* Fig. 1*H*), dots represent single wells tracked, lines represent average for all wells, and error bars are SEM.

Significance for data shown in line plots were tested with pairwise two-way ANOVA with *post hoc* Bonferroni correction for multiple comparisons. Significance for bar and dot graphs was tested using Student's *t* test when single comparisons occurred, and one-way ANOVA with Bonferroni's *post hoc* correction when multiple comparisons occurred. Significance was defined as *p* < 0.05. Statistical analysis was performed using Prism software (https://www.graphpad.com). Note, only wells where all four animals were successfully tracked were included. We excluded individual wells from MWT experiments if we identified technical issues with tracking animals in a given well, such as if only three of four total animals were properly tracked.

### Data availability

Strains and plasmids are available upon request. The authors affirm that all data necessary for confirming the conclusions of the article are present within the article, figures, tables and supplementary materials.

Statistical analysis (Prism) for all quantitative locomotor experiments generated using MWT can be found with the following https://doi.org/10.5061/dryad.x69p8czzd on the Dryad website (https://datadryad.org).

## Supporting information

This article contains supporting information.

## Conflict of interest

B.G. and K.A.M. are equity holders in Evodenovo Inc., which has financial interests in intellectual property associated with opioid research that is not included in this study.

## References

[1] Gonzalez-Hernandez A.J., Munguba H., Levitz J. (2024). Emerging modes of regulation of neuromodulatory G protein-coupled receptors. Trends Neurosci..

[2] Lovinger D.M., Mateo Y., Johnson K.A., Engi S.A., Antonazzo M., Cheer J.F. (2022). Local modulation by presynaptic receptors controls neuronal communication and behaviour. Nat. Rev. Neurosci..

[3] Marder E. (2012). Neuromodulation of neuronal circuits: back to the future. Neuron.

[4] Hooks S.B., Martemyanov K., Zachariou V. (2008). A role of RGS proteins in drug addiction. Biochem. Pharmacol..

[5] Porter M.Y., Koelle M.R. (2009). Insights into RGS protein function from studies in Caenorhabditis elegans. Prog. Mol. Biol. Transl. Sci..

[6] Senese N.B., Kandasamy R., Kochan K.E., Traynor J.R. (2020). Regulator of G-Protein signaling (RGS) protein modulation of opioid receptor signaling as a potential target for pain management. Front. Mol. Neurosci..

[7] Masuho I., Balaji S., Muntean B.S., Skamangas N.K., Chavali S., Tesmer J.J.G. (2020). A global map of G protein signaling regulation by RGS proteins. Cell.

[8] Matthes H.W., Maldonado R., Simonin F., Valverde O., Slowe S., Kitchen I. (1996). Loss of morphine-induced analgesia, reward effect and withdrawal symptoms in mice lacking the mu-opioid-receptor gene. Nature.

[9] Corder G., Tawfik V.L., Wang D., Sypek E.I., Low S.A., Dickinson J.R. (2017). Loss of mu opioid receptor signaling in nociceptors, but not microglia, abrogates morphine tolerance without disrupting analgesia. Nat. Med..

[10] Darcq E., Kieffer B.L. (2018). Opioid receptors: drivers to addiction?. Nat. Rev. Neurosci..

[11] Substance Abuse and Mental Health Services Administration (2020).

[12] Volkow N.D., McLellan A.T. (2016). Opioid abuse in chronic Pain--Misconceptions and mitigation strategies. N. Engl. J. Med..

[13] Hauser A.S., Attwood M.M., Rask-Andersen M., Schioth H.B., Gloriam D.E. (2017). Trends in GPCR drug discovery: new agents, targets and indications. Nat. Rev. Drug Discov..

[14] Connor M., Christie M.D. (1999). Opioid receptor signalling mechanisms. Clin. Exp. Pharmacol. Physiol..

[15] Che T., Roth B.L. (2023). Molecular basis of opioid receptor signaling. Cell.

[16] Hsia J.A., Moss J., Hewlett E.L., Vaughan M. (1984). ADP-ribosylation of adenylate cyclase by pertussis toxin. Effects on inhibitory agonist binding. J. Biol. Chem..

[17] Koehl A., Hu H., Maeda S., Zhang Y., Qu Q., Paggi J.M. (2018). Structure of the micro-opioid receptor-G(i) protein complex. Nature.

[18] Chakrabarti S., Prather P.L., Yu L., Law P.Y., Loh H.H. (1995). Expression of the mu-opioid receptor in CHO cells: ability of mu-opioid ligands to promote alpha-azidoanilido[32P]GTP labeling of multiple G protein alpha subunits. J. Neurochem..

[19] Saidak Z., Blake-Palmer K., Hay D.L., Northup J.K., Glass M. (2006). Differential activation of G-proteins by mu-opioid receptor agonists. Br. J. Pharmacol..

[20] Lamberts J.T., Jutkiewicz E.M., Mortensen R.M., Traynor J.R. (2011). mu-Opioid receptor coupling to Galpha(o) plays an important role in opioid antinociception. Neuropsychopharmacology.

[21] Lamberts J.T., Smith C.E., Li M.H., Ingram S.L., Neubig R.R., Traynor J.R. (2013). Differential control of opioid antinociception to thermal stimuli in a knock-in mouse expressing regulator of G-protein signaling-insensitive Galphao protein. J. Neurosci..

[22] Wang D., Stoveken H.M., Zucca S., Dao M., Orlandi C., Song C. (2019). Genetic behavioral screen identifies an orphan anti-opioid system. Science.

[23] Maza N., Wang D., Kowalski C., Stoveken H.M., Dao M., Sial O.K. (2022). Ptchd1 mediates opioid tolerance via cholesterol-dependent effects on mu-opioid receptor trafficking. Nat. Neurosci..

[24] Kieffer B.L. (2019). An anti-opioid system, courtesy of a worm model. N. Engl. J. Med..

[25] Chronis I.B., Puthenveedu M.A. (2023). Patching holes in the mechanism of opioid tolerance. Trends Pharmacol. Sci..

[26] Koelle M.R. (2018). Neurotransmitter signaling through heterotrimeric G proteins: insights from studies in C. elegans. WormBook.

[27] Hunt T.W., Fields T.A., Casey P.J., Peralta E.G. (1996). RGS10 is a selective activator of G alpha i GTPase activity. Nature.

[28] Watson N., Linder M.E., Druey K.M., Kehrl J.H., Blumer K.J. (1996). RGS family members: GTPase-activating proteins for heterotrimeric G-protein alpha-subunits. Nature.

[29] Saitoh O., Kubo Y., Miyatani Y., Asano T., Nakata H. (1997). RGS8 accelerates G-protein-mediated modulation of K+ currents. Nature.

[30] Tesmer J.J., Berman D.M., Gilman A.G., Sprang S.R. (1997). Structure of RGS4 bound to AlF4--activated G(i alpha1): stabilization of the transition state for GTP hydrolysis. Cell.

[31] Clark M.J., Linderman J.J., Traynor J.R. (2008). Endogenous regulators of G protein signaling differentially modulate full and partial mu-opioid agonists at adenylyl cyclase as predicted by a collision coupling model. Mol. Pharmacol..

[32] Slep K.C., Kercher M.A., Wieland T., Chen C.K., Simon M.I., Sigler P.B. (2008). Molecular architecture of Galphao and the structural basis for RGS16-mediated deactivation. Proc. Natl. Acad. Sci. U. S.A..

[33] Terzi D., Cao Y., Agrimaki I., Martemyanov K.A., Zachariou V. (2012). R7BP modulates opiate analgesia and tolerance but not withdrawal. Neuropsychopharmacology.

[34] Clark M.J., Traynor J.R. (2005). Endogenous regulator of g protein signaling proteins reduce mu-opioid receptor desensitization and down-regulation and adenylyl cyclase tolerance in C6 cells. J. Pharmacol. Exp. Ther..

[35] Zachariou V., Georgescu D., Sanchez N., Rahman Z., DiLeone R., Berton O. (2003). Essential role for RGS9 in opiate action. Proc. Natl. Acad. Sci. U. S. A..

[36] Psifogeorgou K., Papakosta P., Russo S.J., Neve R.L., Kardassis D., Gold S.J. (2007). RGS9-2 is a negative modulator of mu-opioid receptor function. J. Neurochem..

[37] Sutton L.P., Ostrovskaya O., Dao M., Xie K., Orlandi C., Smith R. (2016). Regulator of G-Protein signaling 7 regulates reward behavior by controlling opioid signaling in the striatum. Biol. Psychiatry.

[38] Giles A.C., Desbois M., Opperman K.J., Tavora R., Maroni M.J., Grill B. (2019). A complex containing the O-GlcNAc transferase OGT-1 and the ubiquitin ligase EEL-1 regulates GABA neuron function. J. Biol. Chem..

[39] Cheong M.C., Artyukhin A.B., You Y.J., Avery L. (2015). An opioid-like system regulating feeding behavior in C. elegans. Elife.

[40] Mills H., Ortega A., Law W., Hapiak V., Summers P., Clark T. (2016). Opiates modulate noxious chemical nociception through a complex monoaminergic/peptidergic cascade. J. Neurosci..

[41] Ide S., Kunitomo H., Iino Y., Ikeda K. (2021). Caenorhabditis Elegans exhibits morphine addiction-like behavior via the opioid-like receptor NPR-17. Front. Pharmacol..

[42] Kim A.T., Li S., Kim Y., You Y.J., Park Y. (2024). Food preference-based screening method for identification of effectors of substance use disorders using Caenorhabditis elegans. Life. Sci..

[43] Segalat L., Elkes D.A., Kaplan J.M. (1995). Modulation of serotonin-controlled behaviors by Go in Caenorhabditis elegans. Science.

[44] Wang D., Dao M., Muntean B.S., Giles A.C., Martemyanov K.A., Grill B. (2022). Genetic modeling of GNAO1 disorder delineates mechanisms of Galphao dysfunction. Hum. Mol. Genet..

[45] Anderson G.R., Posokhova E., Martemyanov K.A. (2009). The R7 RGS protein family: multi-subunit regulators of neuronal G protein signaling. Cell. Biochem. Biophys..

[46] Masuho I., Xie K., Martemyanov K.A. (2013). Macromolecular composition dictates receptor and G protein selectivity of regulator of G protein signaling (RGS) 7 and 9-2 protein complexes in living cells. J. Biol. Chem..

[47] Koelle M.R., Horvitz H.R. (1996). EGL-10 regulates G protein signaling in the C. elegans nervous system and shares a conserved domain with many mammalian proteins. Cell.

[48] Patikoglou G.A., Koelle M.R. (2002). An N-terminal region of Caenorhabditis elegans RGS proteins EGL-10 and EAT-16 directs inhibition of G(alpha)o versus G(alpha)q signaling. J. Biol. Chem..

[49] Porter M.Y., Koelle M.R. (2010). RSBP-1 is a membrane-targeting subunit required by the Galpha(q)-specific but not the Galpha(o)-specific R7 regulator of G protein signaling in Caenorhabditis elegans. Mol. Biol. Cell..

[50] Hajdu-Cronin Y.M., Chen W.J., Patikoglou G., Koelle M.R., Sternberg P.W. (1999). Antagonism between G(o)alpha and G(q)alpha in Caenorhabditis elegans: the RGS protein EAT-16 is necessary for G(o)alpha signaling and regulates G(q)alpha activity. Genes. Dev..

[51] Stoveken H.M., Zucca S., Masuho I., Grill B., Martemyanov K.A. (2020). The orphan receptor GPR139 signals via G(q/11) to oppose opioid effects. J. Biol. Chem..

[52] Marwari S., Kowalski C., Martemyanov K.A. (2022). Exploring pharmacological inhibition of G(q/11) as an analgesic strategy. Br. J. Pharmacol..

[53] Robatzek M., Niacaris T., Steger K., Avery L., Thomas J.H. (2001). eat-11 encodes GPB-2, a Gbeta(5) ortholog that interacts with G(o)alpha and G(q)alpha to regulate C. elegans behavior. Curr. Biol..

[54] Doi M., Iwasaki K. (2002). Regulation of retrograde signaling at neuromuscular junctions by the novel C2 domain protein AEX-1. Neuron.

[55] Natochin M., Artemyev N.O. (2003). A point mutation uncouples transducin-alpha from the photoreceptor RGS and effector proteins. J. Neurochem..

[56] Brundage L., Avery L., Katz A., Kim U.J., Mendel J.E., Sternberg P.W. (1996). Mutations in a C. elegans Gqalpha gene disrupt movement, egg laying, and viability. Neuron.

[57] Clark M.J., Furman C.A., Gilson T.D., Traynor J.R. (2006). Comparison of the relative efficacy and potency of mu-opioid agonists to activate Galpha(i/o) proteins containing a pertussis toxin-insensitive mutation. J. Pharmacol. Exp. Ther..

[58] Araldi D., Bonet I.J.M., Green P.G., Levine J.D. (2022). Contribution of G-Protein alpha-Subunits to analgesia, hyperalgesia, and hyperalgesic priming induced by subanalgesic and analgesic doses of fentanyl and morphine. J. Neurosci..

[59] Bastiani C., Mendel J. (2006). Heterotrimeric G proteins in C. elegans. WormBook.

[60] Sanchez G.A., Jutkiewicz E.M., Ingram S., Smrcka A.V. (2022). Coincident regulation of PLCbeta signaling by Gq-Coupled and mu-Opioid receptors opposes opioid-mediated antinociception. Mol. Pharmacol..

[61] Ravi B., Zhao J., Chaudhry S.I., Signorelli R., Bartole M., Kopchock R.J. (2021). Presynaptic Galphao (GOA-1) signals to depress command neuron excitability and allow stretch-dependent modulation of egg laying in Caenorhabditis elegans. Genetics.

[62] Hooks S.B., Waldo G.L., Corbitt J., Bodor E.T., Krumins A.M., Harden T.K. (2003). RGS6, RGS7, RGS9, and RGS11 stimulate GTPase activity of Gi family G-proteins with differential selectivity and maximal activity. J. Biol. Chem..

[63] Miller K.G., Emerson M.D., Rand J.B. (1999). Goalpha and diacylglycerol kinase negatively regulate the Gqalpha pathway in C. elegans. Neuron.

[64] Nurrish S., Segalat L., Kaplan J.M. (1999). Serotonin inhibition of synaptic transmission: Galpha(0) decreases the abundance of UNC-13 at release sites. Neuron.

[65] Wang H., Park H., Liu J., Sternberg P.W. (2018). An efficient genome editing strategy to generate putative null mutants in Caenorhabditis elegans using CRISPR/Cas9. G3 (Bethesda).

[66] Burns A.R., Wallace I.M., Wildenhain J., Tyers M., Giaever G., Bader G.D. (2010). A predictive model for drug bioaccumulation and bioactivity in Caenorhabditis elegans. Nat. Chem. Biol..

[67] Lindblom T.H., Dodd A.K. (2006). Xenobiotic detoxification in the nematode Caenorhabditis elegans. J. Exp. Zool. A. Comp. Exp. Biol..

[68] Broeks A., Gerrard B., Allikmets R., Dean M., Plasterk R.H. (1996). Homologues of the human multidrug resistance genes MRP and MDR contribute to heavy metal resistance in the soil nematode Caenorhabditis elegans. EMBO. J..

[69] Broeks A., Janssen H.W., Calafat J., Plasterk R.H. (1995). A P-glycoprotein protects Caenorhabditis elegans against natural toxins. EMBO. J..

[70] James C.E., Davey M.W. (2009). Increased expression of ABC transport proteins is associated with ivermectin resistance in the model nematode Caenorhabditis elegans. Int. J. Parasitol..

[71] O'Reilly L.P., Luke C.J., Perlmutter D.H., Silverman G.A., Pak S.C. (2014). C. elegans in high-throughput drug discovery. Adv. Drug. Deliv. Rev..

