# Supplementary material for: G protein regulatory network shapes magnitude and kinetics of behavioral responses in an engineered opioid receptor model
Source: J Biol Chem. 2026 Apr 2;302(5):111425. doi: 10.1016/j.jbc.2026.111425 (PMC13157061; doi:10.1016/j.jbc.2026.111425)
Supplement: Figures S1–S6 and Tables S1–S4 [file mmc1.docx]

**Supplemental Materials (Supplemental Figures, Movie Legends and Supplemental Tables)**

**G protein regulatory network shapes magnitude and kinetics of behavioral responses in an engineered opioid receptor model**

Deziree L. Coleman^1,2,3*^, Rachel J. Ren^1*^, Karla J. Opperman^1^, Elizabeth X. Kwan^1^, Kirill A. Martemyanov^4^, and Brock Grill^1,2,3 #^

^1^ Norcliffe Foundation Center for Integrative Brain Research, Seattle Children’s Research Institute, Seattle Children’s Hospital, Seattle, WA

^2^ Department of Pharmacology, University of Washington School of Medicine, Seattle, WA

^3^ Department of Pediatrics, University of Washington School of Medicine, Seattle, WA

^4^ Department of Physiology and Biophysics, University of Miami Medical School, Miam, FL, USA

* both authors contributed equally

^#^ correspondence: brock.grill@seattlechildrens.org


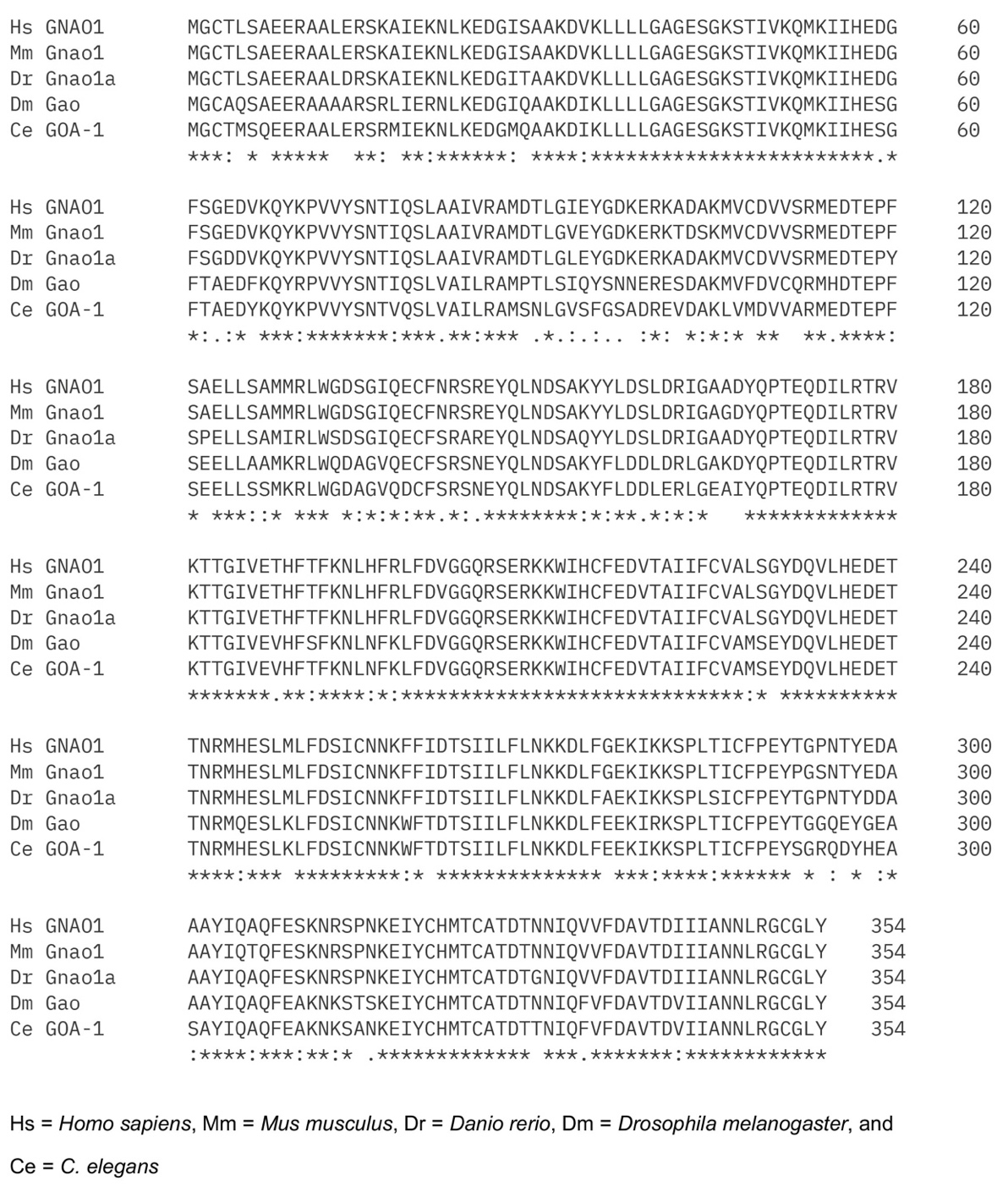
**Supplemental Figure 1. Gαo** **conservation across species.**

Clustal Omega alignment of full-length Gαo protein sequences from multiple species. Shown are sequence alignments between Gαo from human (Hs GNAO1), mouse (Mm Gnao1), zebrafish (Dr Gnao1a), Drosophila (Dm Gao), and *C. elegans* (Ce GOA-1). Annotated are identical residues (*) highly conserved residues (:) and mildly conserved residues (.).

**
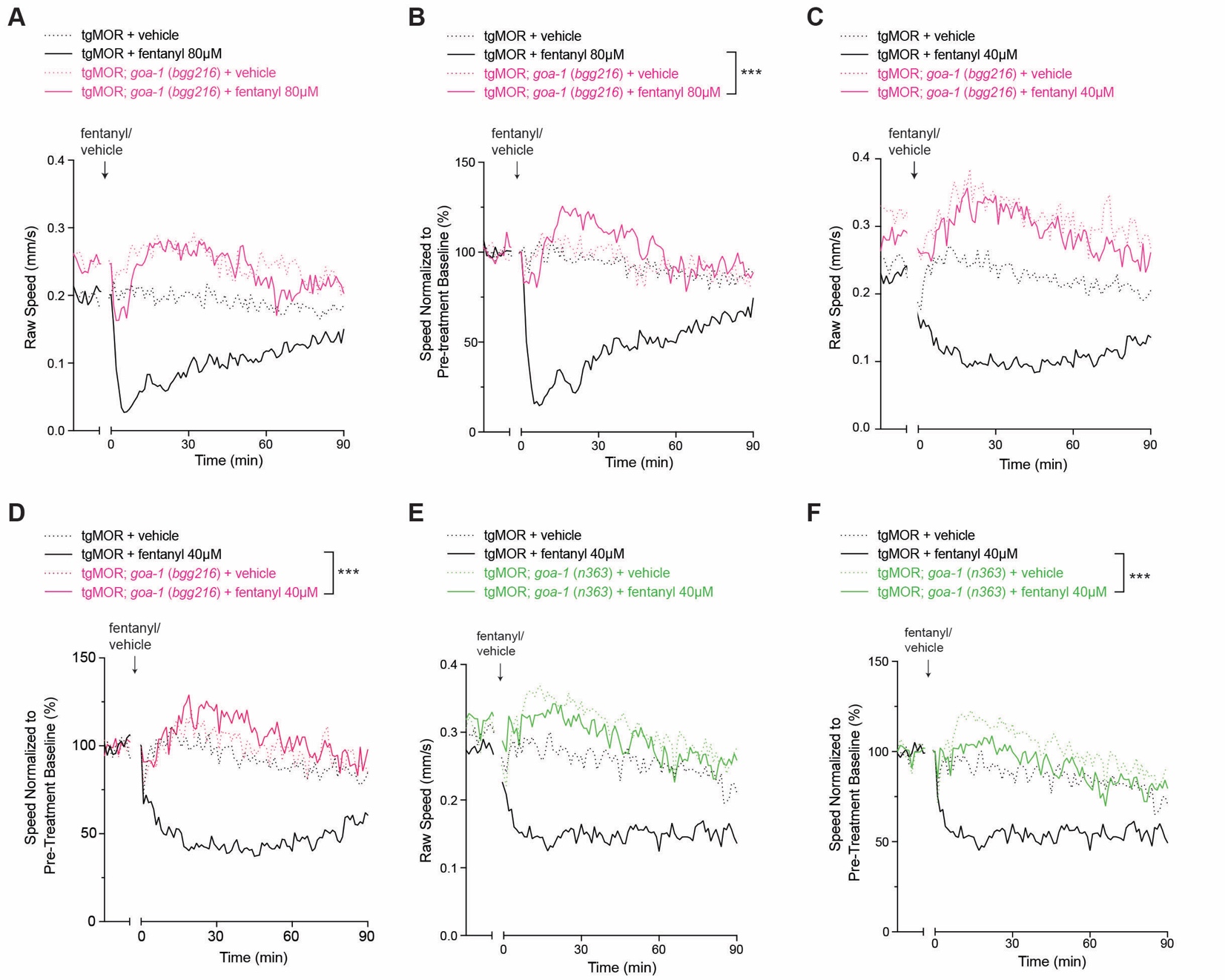
Supplemental Figure 2**. **Expanded analysis of MWT data showing GOA-1 Gαo** **mediates effects of fentanyl on tgMOR *C. elegans*.**

***A-B)*** Quantitation of ***A)*** raw locomotor speed and ***B)*** speed normalized to pre-treatment baseline locomotion shows tgMOR; *goa-1 (bgg216)* mutants have reduced sensitivity to 80µM fentanyl. Arrow indicates fentanyl or vehicle application. ***C-D)*** Quantitation of ***C)*** raw locomotor speed and ***D)*** speed normalized to pre-treatment baseline indicates tgMOR; *goa-1 (bgg216)* mutants have reduced responses to 40µM fentanyl. ***E-F)*** Quantitation of ***E)*** raw locomotor speed and ***F)*** speed normalized to pre-treatment baseline shows tgMOR; *goa-1* shows tgMOR; *goa-1* (*n363*) mutants have reduced responses to 40µM fentanyl. **For A-F**, solid lines in plots represent mean speed of tracked animals (4 animals/well, 5 wells per genotype per experiment and 3-4 independent experiments for all genotypes and treatments). **For B, D, and F**, significance for genotype annotations in plots tested using two-way ANOVA with post-hoc Bonferroni correction for multiple comparisons. ****P*<0.001

**
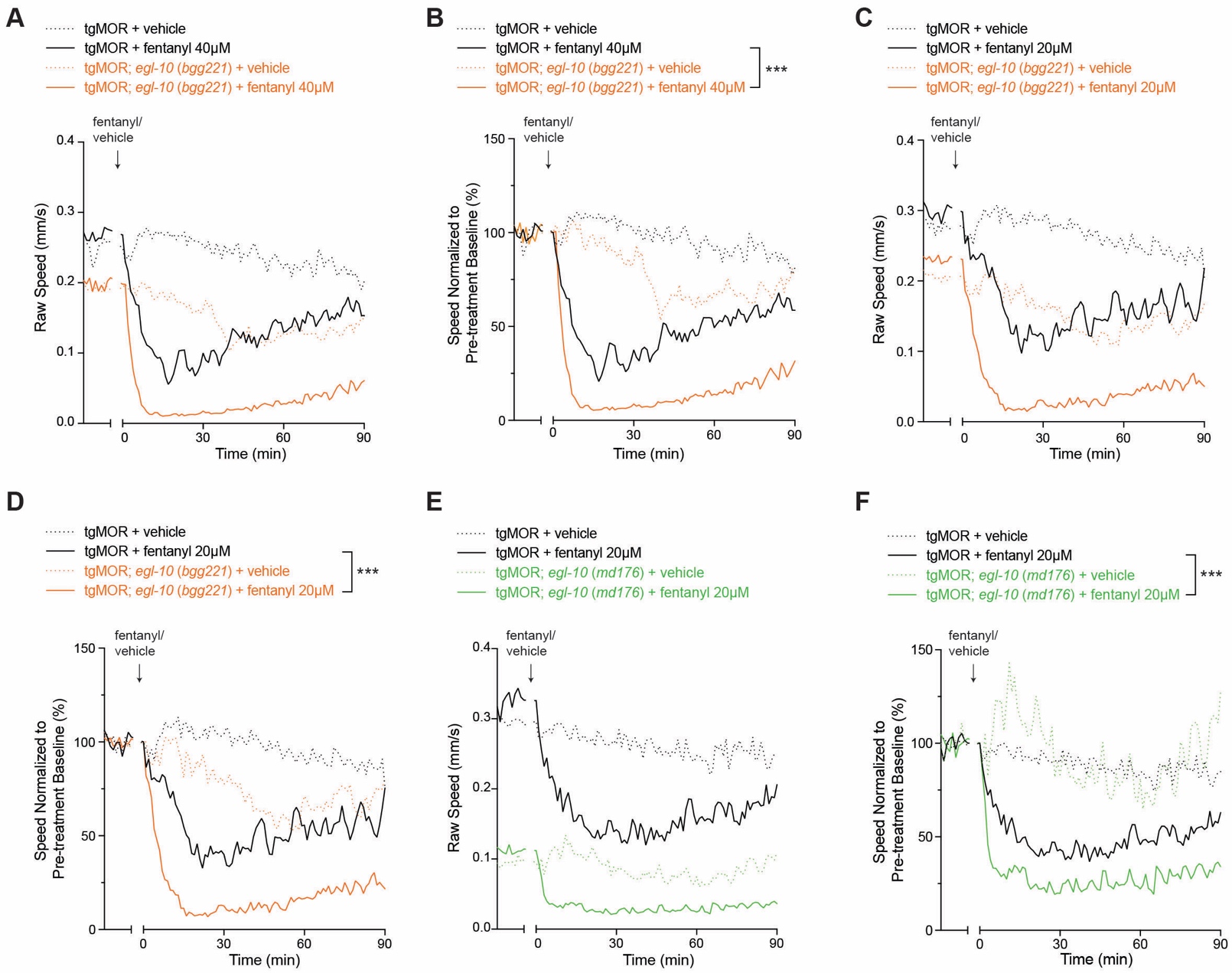
Supplemental Figure 3**. **Expanded analysis of MWT data for tgMOR; *egl-10* mutants.**

***A-B)*** Quantitation of ***A)*** raw locomotor speed and ***B)*** speed normalized to pre-treatment baseline locomotion shows tgMOR; *egl-10* (*bgg221*) mutants have increased sensitivity to 40µM fentanyl. Arrow indicates fentanyl or vehicle application. ***C-D)*** Quantitation of ***C)*** raw locomotor speed and ***D)*** speed normalized to pre-treatment baseline indicates tgMOR; *egl-10* (*bgg221*) mutants have increased responses to 20µM fentanyl. ***E-F)*** Quantitation of ***E)*** raw locomotor speed and ***F)*** speed normalized to pre-treatment baseline shows tgMOR; *egl-10* (*md176*) mutants have increased responses to 20µM fentanyl. **For A-F**, solid lines in plots represent mean speed of tracked animals (4 animals/well, 5 wells per genotype per experiment and 3-4 independent experiments for all genotypes and treatments). **For B, D, and F**, significance for genotype annotations in plots tested using two-way ANOVA with post-hoc Bonferroni correction for multiple comparisons. ****P*<0.001

**
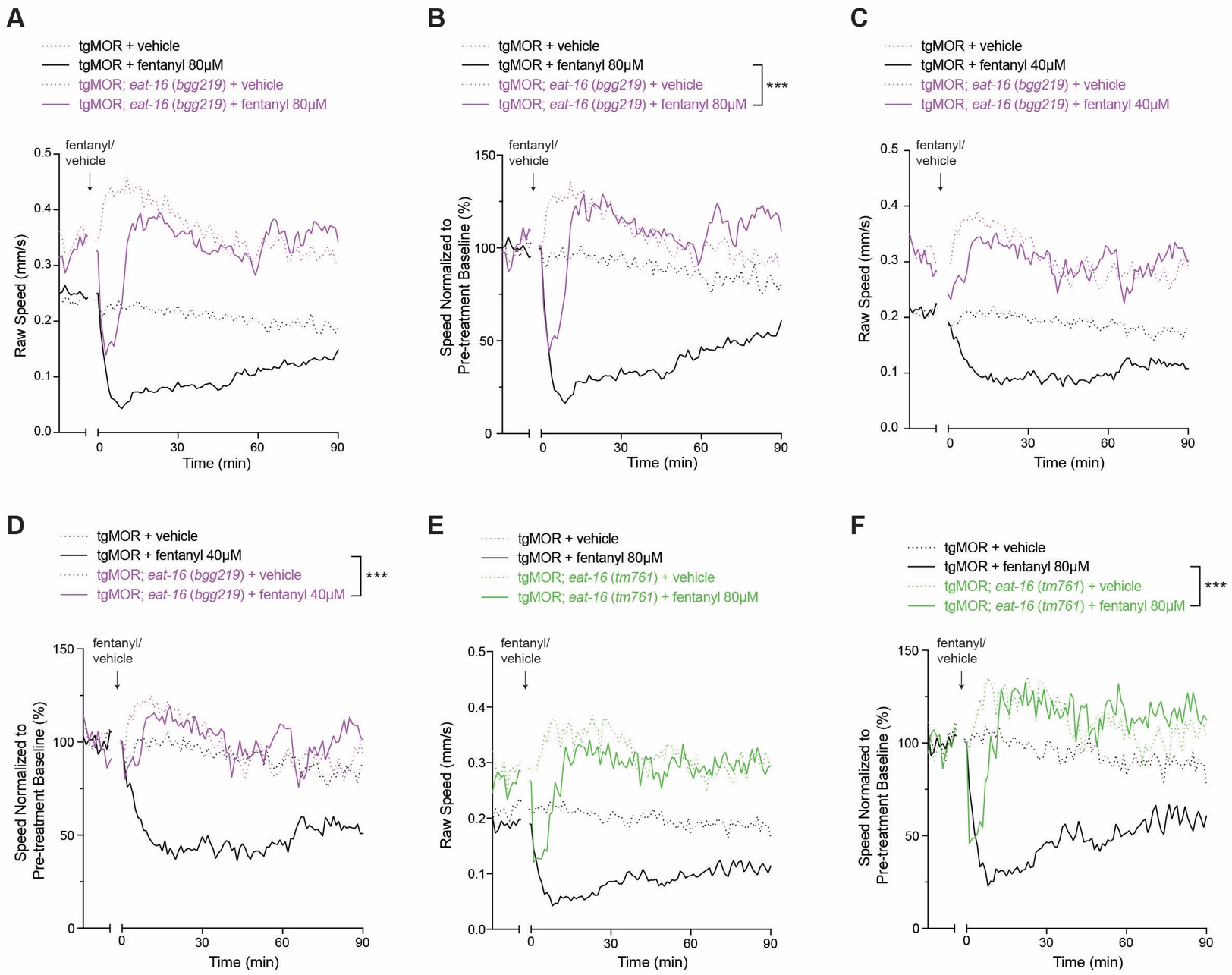
**

**Supplemental Figure 4**. **Expanded analysis of MWT data for tgMOR; *eat-16* mutants.**

***A-B)*** Quantitation of ***A)*** raw locomotor speed and ***B)*** speed normalized to pre-treatment baseline locomotion shows tgMOR; *eat-16* (*bgg219*) mutants have increased sensitivity in time of response to 80µM fentanyl as well as reduced amplitude of response. Arrow indicates fentanyl or vehicle application. ***C-D)*** Quantitation of ***C)*** raw locomotor speed and ***D)*** speed normalized to pre-treatment baseline indicates tgMOR; *eat-16* (*bgg219*) mutants have increased sensitivity in time of response to 40µM fentanyl as well as reduced amplitude of response. ***E-F)*** Quantitation of ***E)*** raw locomotor speed and ***F)*** speed normalized to pre-treatment baseline shows tgMOR; *eat-16* (*tm761*) mutants have increased sensitivity in time of response to 80µM fentanyl as well as reduced amplitude of response. **For A-F**, solid lines in plots represent mean speed of tracked animals (4 animals/well, 5 wells per genotype per experiment and 3-5 independent experiments for all genotypes and treatments). **For B, D, and F**, significance for genotype annotations in plots tested using two-way ANOVA with post-hoc Bonferroni correction for multiple comparisons. ****P*<0.001

**
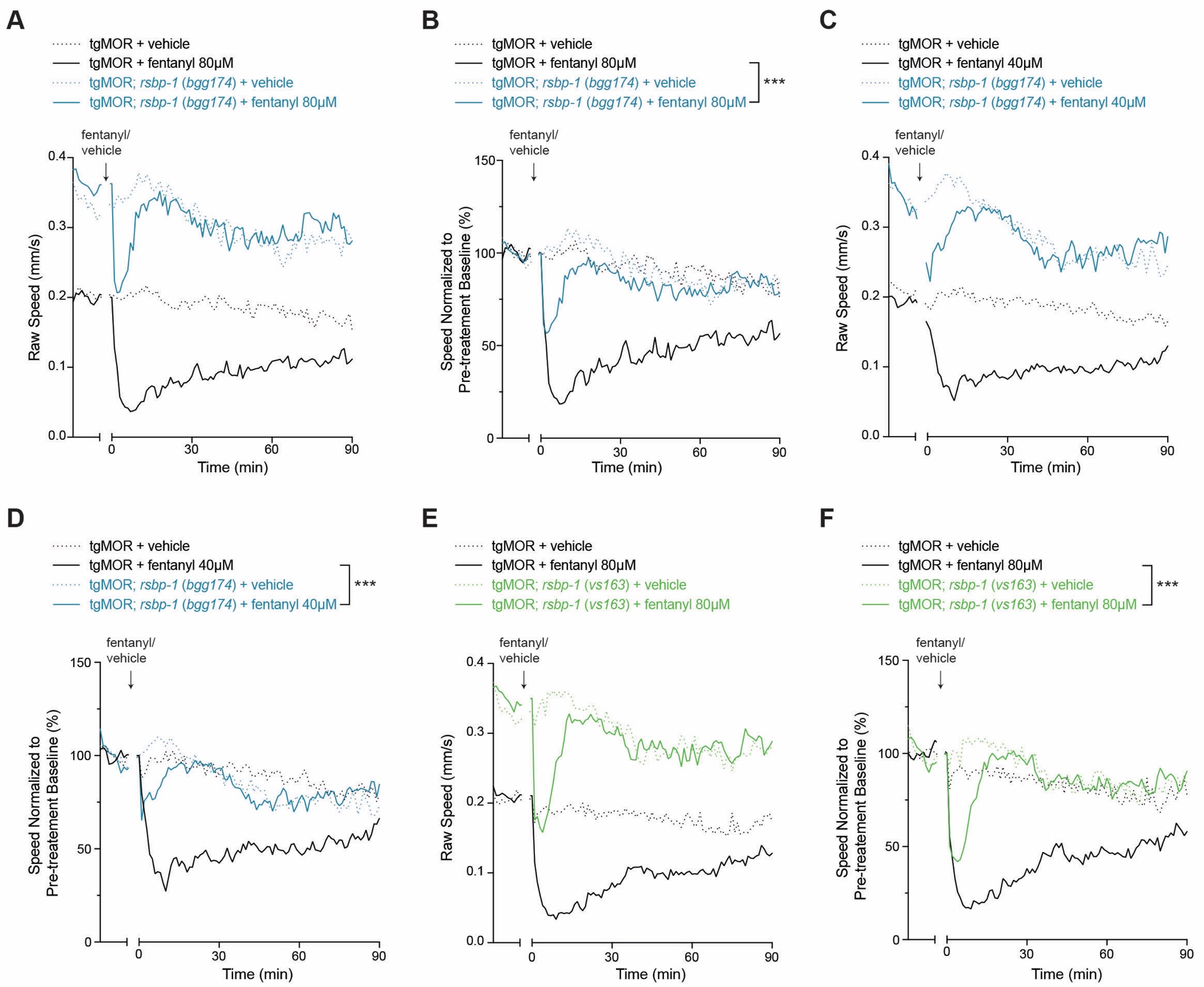
**

**Supplemental Figure 5**. **Expanded analysis of MWT data for tgMOR; *rsbp-1* mutants.**

***A-B)*** Quantitation of ***A)*** raw locomotor speed and ***B)*** speed normalized to pre-treatment baseline locomotion shows tgMOR; *rsbp-1* (*bgg174*) mutants have increased sensitivity in time of response to 80µM fentanyl as well as reduced amplitude of response. Arrow indicates fentanyl or vehicle application. ***C-D)*** Quantitation of ***C)*** raw locomotor speed and ***D)*** speed normalized to pre-treatment baseline indicates tgMOR; *rsbp-1* (*bgg174*) mutants have increased sensitivity in time of response to 40µM fentanyl as well as reduced amplitude of response. ***E-F)*** Quantitation of ***E)*** raw locomotor speed and ***F)*** speed normalized to pre-treatment baseline shows tgMOR; *rsbp-1* (*vs163*) mutants have increased sensitivity in time of response to 80µM fentanyl as well as reduced amplitude of response. **For A-F**, solid lines in plots represent mean speed of tracked animals (4 animals/well, 5 wells per genotype per experiment and 4 independent experiments for all genotypes and treatments). **For B, D, and F**, significance for genotype annotations in plots tested using two-way ANOVA with post-hoc Bonferroni correction for multiple comparisons. ****P*<0.001

**
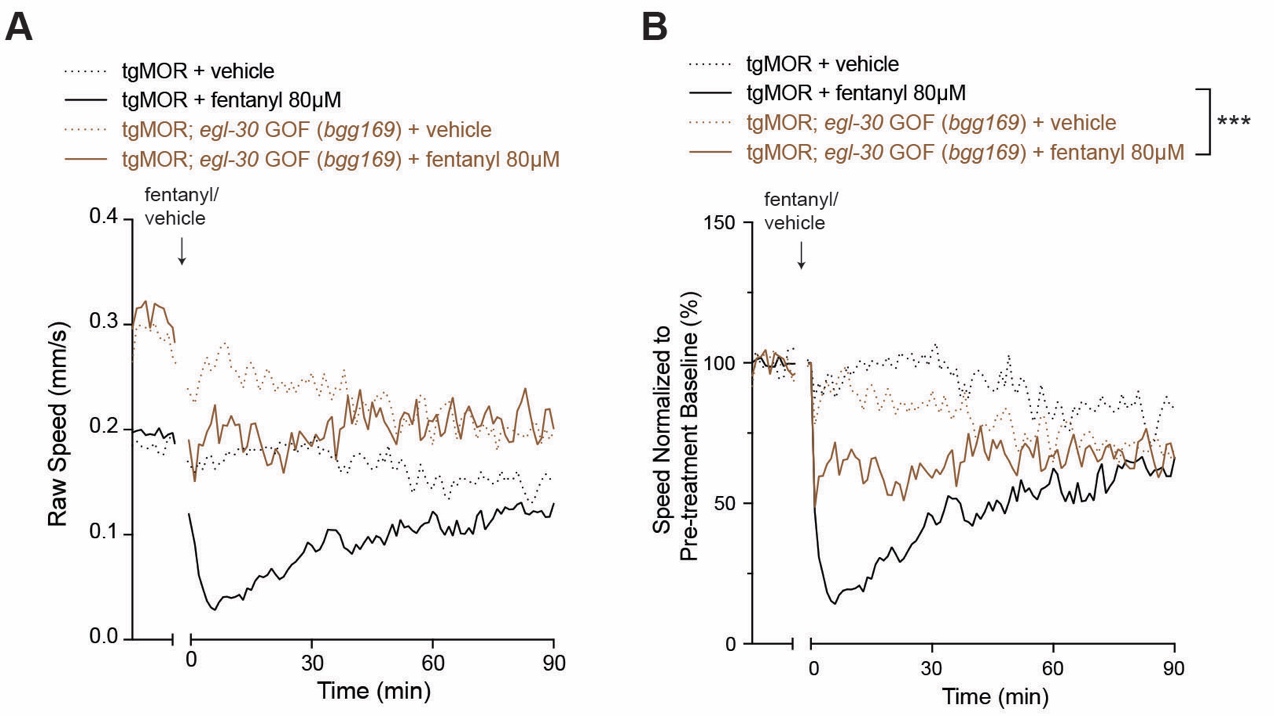
**

**Supplemental Figure 6**. **Expanded analysis of MWT data for tgMOR; *egl-30* mutants.**

***A-B)*** Quantitation of ***A)*** raw locomotor speed and ***B)*** speed normalized to pre-treatment baseline locomotion shows tgMOR; *egl-30* (*bgg169*) mutants have decreased sensitivity to 80µM fentanyl. Arrow indicates fentanyl or vehicle application. **For A and B**, solid lines in plots represent mean speed of tracked animals (4 animals/well, 5 wells per genotype per experiment and 4 independent experiments for all genotypes and treatments). **For B**, Significance for genotype annotations in plots was tested using two-way ANOVA with post-hoc Bonferroni correction for multiple comparisons. ****P*<0.001

**Supplemental Movie Legends**

**Supplemental Movie 1: Example showing tgMOR *C. elegans* treated with vehicle locomote rapidly and show increased swimming in response to touch stimulus.** 15-second movie showing tgMOR *C. elegans* treated with vehicle for 10 minutes display high-intensity swimming locomotor behavior in liquid that is stimulated by contact with cat whisker.

**Supplemental Movie 2: Example showing tgMOR *C. elegans* treated with fentanyl do not locomote in liquid and do not respond to touch stimulus.** 15-second movie showing tgMOR *C. elegans* treated with 80µM fentanyl for 10 minutes are paralyzed in liquid and do not respond to stimulation with cat whisker.

**Supplemental Movie 3: Example showing tgMOR; *goa-1* (*bgg216*) mutants treated with vehicle locomote rapidly in liquid.** 20-second movie showing tgMOR (left) and tgMOR; *goa-1* (*bgg216*) mutants (right) treated with vehicle for 10 minutes display high-intensity locomotor behavior.

**Supplemental Movie 4**. **Example showing** **tgMOR; *goa-1* (*bgg216*) mutants display reduced sensitivity to fentanyl.** 20-second movie shot 10 minutes after 80µM fentanyl treatment shows tgMOR animals (left) have reduced locomotor activity, while tgMOR; *goa-1* (*bgg216*) mutants (right) are not affected by fentanyl.

**Supplemental Movie 5: Example showing tgMOR; *egl-10* (*bgg221*) mutants treated with vehicle locomote rapidly in liquid.** 20-second movie showing tgMOR (left) and tgMOR; *egl-10* (*bgg221*) mutants (right) treated with vehicle for 9 minutes display high-intensity locomotor behavior.

**Supplemental Movie 6: Example showing tgMOR; *egl-10* (*bgg221*) mutants display increased sensitivity to fentanyl.** 20-second movie shot 8 minutes after 40µM fentanyl treatment shows tgMOR; *egl-10 (bgg221)* mutants (right) have greater reductions in locomotor speed than tgMOR animals (left).

**Supplementary Table 1: Transgenic and CRISPR Strains**

| **Figure** | **Strain** | **Genotype** |
| --- | --- | --- |
| Figs 1-6  Supp Figs 2-6 | XMN1408 | *bggIs79* [P_rgef-1_FLAG mouse MOR; P_ttx-3_RFP] X |
| Fig 1 | N2 | wildtype |
| Fig 1 | XMN1587 | *npr-17*(*bgg329* [3-frame stop cassette CRISPR]) III; *bggIs79* [P_rgef-1_FLAG mouse MOR; P_ttx-3_RFP] X |
| Fig 2  Supp Fig 2 | XMN1582 | *goa-1*(*bgg216* [L81fs CRISPR]) I; *bggIs79* [P_rgef-1_FLAG mouse MOR; P_ttx-3_RFP] X |
| Fig 2  Supp Fig 2 | XMN1391 | *goa-1*(*n363*) I; *bggIs79* [P_rgef-1_FLAG mouse MOR; P_ttx-3_RFP] X |
| Fig 3  Supp Fig 3 | XMN1585 | *egl-10*(*bgg221* [3-frame stop cassette CRISPR]) V; *bggIs79* [P_rgef-1_FLAG mouse MOR; P_ttx-3_RFP] X |
| Fig 3  Supp Fig 3 | XMN1586 | *egl-10(md176)* V*; bggIs79* [P_rgef-1_FLAG mouse MOR; P_ttx-3_RFP] X |
| Fig 4  Supp Fig 4 | XMN1583 | *eat-16*(*bgg219* [Y109Stop CRISPR]) I; *bggIs79* [P_rgef-1_FLAG mouse MOR; P_ttx-3_RFP] X |
| Fig 4  Supp Fig 4 | XMN1584 | *eat-16*(*tm761*) I; *bggIs79* [P_rgef-1_FLAG mouse MOR; P_ttx-3_RFP] X |
| Fig 5  Supp Fig 5 | XMN1581 | *rsbp-1*(*bgg174* [Q31Stop CRISPR]) I; *bggIs79* [P_rgef-1_FLAG mouse MOR; P_ttx-3_RFP] X |
| Fig 5  Supp Fig 5 | XMN1409 | *rsbp-1*(*vs163*) I; *bggIs79* [P_rgef-1_FLAG mouse MOR; P_ttx-3_RFP] X |
| Fig 6  Supp Fig 6 | XMN1428 | *egl-30*(*bgg169* [R243Q CRISPR]) I; *bggIs79* [P_rgef-1_FLAG mouse MOR; P_ttx-3_RFP] X |

**Supplementary Table 2: Genotyping primers**

| **Gene** | **Allele/ Construct** | **Primer Sequence** |
| --- | --- | --- |
| *npr-17* | *bgg328* | *bgg328* fwd: 5’ cccaaaccagattcagacaa 3’  *bgg328* rev: 5’ cgattggtttttggaaagtt 3’ |
| *goa-1* | *bgg216* | *bgg216* fwd: 5’ AAACTGCTGCTACTTGGTGC 3’  *bgg216* rev: 5’ ggcacgaaattttcaagtgatcc 3’  digest: HpyCH4V |
| *goa-1* | *n363* | common fwd: 5’ agcgattggtaggctttttg 3’  wt rev: 5’ gttacagcatggtggccttt 3’  *n363* rev: 5’ gagctgtgaaaattcgcaaa 3’ |
| *egl-10* | *bgg221* | *bgg221* fwd: 5’ cttcccaagtgcatctcaaaag 3’  *bgg221* rev: 5’ CATCTGGCTCATCTGATCGC 3’ |
| *egl-10* | *md176* | Not PCR genotyped. Genotyped by egg laying phenotype. |
| *eat-16* | *bgg219* | *bgg219* fwd: 5’ GCCAACCTTTCAAAGACGATG 3’  *bgg219* rev: 5’ aatagctttcgactccaaaactg 3’  digest: MseI |
| *eat-16* | *tm761* | *tm761* fwd: 5’ taacagGGTGCTCCTGACG 3’  *tm761* rev: 5’ GCAAAGgcaagattttccag 3’ |
| *rsbp-1* | *bgg174* | *bgg174* fwd: 5’ gaaacaattatctcccaaaggcc 3’  *bgg174* rev: 5’ CGCAGTTAAGGGCAGATGgt 3’  digest: AluI |
| *rsbp-1* | *vs163* | *vs163* fwd: 5’ AAATCCCTCCATTCCGATCAG 3’  *vs163* rev: 5’ GCAGTTAAGGGCAGATGgtaa 3’ |
| *egl-30* | *bgg169* | *bgg169* fwd: 5’ ggggatgagcaaaacacagg 3’  *bgg169* rev: 5’ ctagaattgagccgcgacac 3’  digest: BccI |

**Supplementary Table 3: CRISPR reagents**

| **Gene** | **Construct** | **crRNA Target Sequence + PAM** | **Repair Template** |
| --- | --- | --- | --- |
| *npr-17* | *npr-17*  3-frame stop cassette (*bgg328*) | TGTAGATCTTCGTGATTGGG**AGG** | AGTACAATATAGTGAATGATGTTCTACCT**CCT**CCCGGGAAGTTTGTCCAGAGCAGAGGTGACTAAGTGATAAGCTAGCAATCACGAAGATCTACATGTTGTAATAATGGCAGT (reverse complement) |
| *goa-1* | *goa-1* L81fs  (*bgg216*) | CAGTAACACGGTTCAATCA**TGG** | GACTACAAACAGTACAAGCCGGTTGTCTACAGTAATACAGTGCAATCT**TGG**TCGCTATTTTGCGAGCCATGAGCAACTTAGGCG |
| *egl-10* | *egl-10*  3-frame stop cassette (*bgg221*) | TCTTGTACATCCAAACCACA**TGG** | TGCAAGCAACGAGGAGCGTCTTGTACATCCAAACCGGGAAGTTTGTCCAGAGCAGAGGTGACTAAGTGATAAGCTAGCACA**TGG**TGTACCGTAAGATGGAGATGCTTGTCAAT |
| *eat-16* | *eat-16* Y109Stop  (*bgg219*) | CAATCGCTTGTTGAGGTAAA**TGG** | gaaaagttattgtatctgatccactatatttcag**CCA**TTTAGTTAAATAAGCGATTGCTCCGAAATGAGCAAAAACACGGGTT (reverse complement) |
| *rsbp-1* | *rsbp-1* Q31Stop  (*bgg174*) | CTCTTCCGTGTAGCAACACA**AGG** | TGGTGCACGAGTGCAACGTGCAACTTGCACTCTTCAGAGTAGCTACTTA**AGG**AATCGGAACCGCCCAAGATGGTGCATCATTA |
| *egl-30* | *egl-30* R243Q GOF (*bgg169*) | GAGCTTTCGATTCTTCCATT**CGG** | aaaaaaaaaacaaactagaaatcaatcttgcagAATCAGATGGAAGAATCGAAAGCTCTGTTCCGAACGATCATCA |

Legend: underline (crRNA targeting sequence), **bold** (Pam sequence), Green: insertions, Red: Substitutions, Blue: silent mutations in repair to prevent Cas9 re-cutting.

**Supplementary Table 4: Injection conditions**

| **Figure** | **Gene edit** | **Injected Strain** | **Injection Mix** |
| --- | --- | --- | --- |
| Fig 1 | *npr-17* 3-frame stop cassette (*bgg328*) | *bggIs79* | 5.0 uL Cas9 (10 μg/μl) |
|  |  |  | 5.0 uL IDT tracrRNA (4 μg/μl) in Duplex Buffer |
|  |  |  | 0.56 ul *dpy-10* crRNA (4 μg/μl) |
|  |  |  | 2.0 ul *npr-17* crRNA (4 μg/μl) |
|  |  |  | 2.2 ul *npr-17* repair oligo (1 μg/μl) |
|  |  |  | 0.55 μl *dpy-10* repair ssODN, 500 ng/μl |
|  |  |  | 4.69 μl H_2_O |
| Fig 2  Supp Fig 2 | *goa-1* L81fs (*bgg216*) | *bggIs79* | 5.0 uL Cas9 (10 μg/μl) |
|  |  |  | 5.0 uL IDT tracrRNA (4 μg/μl) in Duplex Buffer |
|  |  |  | 0.56 ul *dpy-10* crRNA (4 μg/μl) |
|  |  |  | 2.0 ul *goa*-1 crRNA (4 μg/μl) |
|  |  |  | 2.2 ul *goa*-1 repair oligo (1 μg/μl) |
|  |  |  | 0.55 μl *dpy-10* repair ssODN, 500 ng/μl |
|  |  |  | 4.69 μl H_2_O |
| Fig 3,  Supp Fig 3 | *egl-10* 3-frame stop cassette (*bgg221*) | *bggIs79* | 5.0 uL Cas9 (10 μg/μl) |
|  |  |  | 5.0 uL IDT tracrRNA (4 μg/μl) in Duplex Buffer |
|  |  |  | 0.56 ul *dpy-10* crRNA (4 μg/μl) |
|  |  |  | 2.0 ul *egl-10* crRNA (4 μg/μl) |
|  |  |  | 2.2 ul *egl-10* repair oligo (1 μg/μl) |
|  |  |  | 0.55 μl *dpy-10* repair ssODN, 500 ng/μl |
|  |  |  | 4.69 μl H_2_O |
| Fig 4,  Supp Fig 4 | *Eat-16* Y109Stop (*bgg219*) | *bggIs79* | 5.0 uL Cas9 (10 μg/μl) |
|  |  |  | 5.0 uL IDT tracrRNA (4 μg/μl) in Duplex Buffer |
|  |  |  | 0.56 ul *dpy-10* crRNA (4 μg/μl) |
|  |  |  | 2.0 ul *eat-16* crRNA (4 μg/μl) |
|  |  |  | 2.2 ul *eat-16* repair oligo (1 μg/μl) |
|  |  |  | 0.55 μl *dpy-10* repair ssODN, 500 ng/μl |
|  |  |  | 4.69 μl H_2_O |
| Fig 5,  Supp Fig 5 | *rsbp-1* Q31Stop  (*bgg174*) | *bggIs79* | 5.0 uL Cas9 (10 μg/μl) |
|  |  |  | 5.0 uL IDT tracrRNA (4 μg/μl) in Duplex Buffer |
|  |  |  | 0.56 ul *dpy-10* crRNA (4 μg/μl) |
|  |  |  | 2.0 ul *rsbp-1* crRNA (4 μg/μl) |
|  |  |  | 2.2 ul *rsbp*-1 repair oligo (1 μg/μl) |
|  |  |  | 0.55 μl *dpy-10* repair ssODN, 500 ng/μl |
|  |  |  | 4.69 μl H_2_O |
| Fig 6,  Supp Fig 6 | *egl-30* R243Q GOF (*bgg169*) | *bggIs79* | 5.0 uL Cas9 (10 μg/μl) |
|  |  |  | 5.0 uL IDT tracrRNA (4 μg/μl) in Duplex Buffer |
|  |  |  | 0.56 ul *dpy-10* crRNA (4 μg/μl) |
|  |  |  | 2.0 ul *egl-30* crRNA (4 μg/μl) |
|  |  |  | 2.2 ul *egl-30* repair oligo (1 μg/μl) |
|  |  |  | 0.55 μl *dpy-10* repair ssODN, 500 ng/μl |
|  |  |  | 4.69 μl H_2_O |
